# Global, Survival, and Apoptotic Transcriptome during Mouse and Human Early Embryonic Development

**DOI:** 10.1155/2018/5895628

**Published:** 2018-11-01

**Authors:** D. Haouzi, I. Boumela, K. Chebli, S. Hamamah

**Affiliations:** ^1^CHU Montpellier, Institut de Médecine Régénératrice et de Biothérapie, Hôpital Saint-Eloi, Montpellier 34295, France; ^2^INSERM U1203, Hôpital Saint-Eloi, Montpellier 34295, France; ^3^UMR 5535 IGMM, Equipe “Métabolisme des ARNs”, Montpellier 34090, France; ^4^Université de Montpellier, UFR de Médecine, Equipe “Développement Embryonnaire Précoce Humain et Pluripotence”, Montpellier 34093, France; ^5^CHU Montpellier, ART/PGD Division, Département de Biologie de la Reproduction, Hôpital Arnaud de Villeneuve, Montpellier 34295, France

## Abstract

Survival and cell death signals are crucial for mammalian embryo preimplantation development. However, the knowledge on the molecular mechanisms underlying their regulation is still limited. Mouse studies are widely used to understand preimplantation embryo development, but extrapolation of these results to humans is questionable. Therefore, we wanted to analyse the global expression profiles during early mouse and human development with a special focus on genes involved in the regulation of the apoptotic and survival pathways. We used DNA microarray technology to analyse the global gene expression profiles of preimplantation human and mouse embryos (metaphase II oocytes, embryos at the embryonic genome activation stage, and blastocysts). Components of the major apoptotic and survival signalling pathways were expressed during early human and mouse embryonic development; however, most expression profiles were species-specific. Particularly, the expression of genes encoding components and regulators of the apoptotic machinery were extremely stable in mouse embryos at all analysed stages, while it was more stage-specific in human embryos.* CASP3*,* CASP9*, and* AIF* were the only apoptosis-related genes expressed in both species and at all studied stages. Moreover, numerous transcripts related to the apoptotic and survival pathway were reported for the first time such as* CASP6* and* IL1RAPL1* that were specific to MII oocytes;* CASP2*,* ENDOG*, and* GFER* to blastocysts in human. These findings open new perspectives for the characterization and understanding of the survival and apoptotic signalling pathways that control early human and mouse embryonic development.

## 1. Introduction

The ability of early mammalian embryos to cope with stress during the first stages of development could be controlled by the activation of survival pathways through autocrine and paracrine regulatory signals [1], and also by the establishment of a cell death program to ensure the elimination of damaged cells [2, 3]. Apoptosis has been described in human and animal oocytes and early embryos* in vitro* and* in vivo* [4, 5]. However, sensitivity to apoptosis appears to be developmentally regulated [6], suggesting that a fine balance between apoptotic and survival signals is established in preimplantation embryos. Indeed, in many mammalian species including humans, apoptosis is first observed after embryonic genome activation (EGA) and is common at the blastocyst stage [5, 7, 8]. Although apoptosis extent and timing during preimplantation development are likely to be critical for embryo development, our knowledge on the causes, roles, and molecular mechanisms that underlie embryo death and survival before implantation are still very limited. Our group showed that some genes implicated in the apoptotic machinery are expressed in human and animal oocytes and early embryos [3, 9]. However, apoptosis is a highly coordinated, multistep process that requires the actions and interactions of more than 500 gene products [10]. Therefore, to better understand these processes we need to characterize the global expression pattern of apoptosis and survival regulatory factors during early embryo development.

To overcome the ethical and practical concerns that limit research on human embryos, most groups have been using animal models, particularly mice, to study cell death and survival in preimplantation embryos. However, species-specific differences, such as EGA timing and gene expression profiles, could limit the extrapolation of the results obtained in mouse embryos to human embryos.

In the present study, we used DNA microarrays to compare the global transcriptome during early human and mouse embryonic development with a special focus on genes involved in the regulation of the apoptotic and survival pathways.

## 2. Materials and Methods

### 2.1. Human Samples

#### 2.1.1. Patients' Characteristics

Patients (n=47) referred for conventional IVF or intracytoplasmic sperm injection (ICSI) were recruited for this study after signature of the written informed consent between January 2009 to December 20012. The part of this project on human embryos was approved by the French National Agency of Biomedicine (N°AFSB12002255) for human embryo research. All patients had normal serum FSH, LH, and estradiol at day 3 of controlled ovarian stimulation (COS). After COS, cumulus-oocyte complexes were collected by vaginal puncture under ultrasound guidance 35-36h after administration of 5000 IU of human chorionic gonadotrophin (hCG). Oocytes were denuded of cumulus cells by enzymatic treatment with 80 UI/ml hyaluronidase solution (SynVitro®Hyadase, MediCult) to assess nuclear maturity for ICSI.

#### 2.1.2. Oocytes

Unfertilized metaphase II (MII) oocytes were collected 24h after sperm microinjection as previously described [11]. Pools (n=4) of 12, 19, 23, and 24 mature unfertilized MII oocytes obtained from 39 patients (age mean ± SD: 37 ± 4.3 yrs) were used for microarray analyses, and four single mature MII oocytes from two patients (age mean± SD: 35 ± 3.2 yrs) were used for RT-qPCR validation (Table 1). Oocytes were directly placed in RLT RNA extraction buffer (RNeasy Micro kit, Qiagen, Valencia, CA, USA) and frozen at -80°C before total RNA extraction.

#### 2.1.3. Day 3 Cleavage Embryos and Day 5/6 Blastocysts

Three days after fertilization, supernumerary embryos (n=13) obtained from six patients were placed in RLT RNA extraction buffer or cultured in G-2 PLUS medium (Vitrolife) until blastocyst stage (day 5/6 after fertilization) (Table 1). Day 3 embryos were all at the 6-8 cell stage with <20% fragmentation. According to Gardner's classification, the eight used blastocysts were grade B1/3CC/4CC/5CC [12].

Individual day 3 embryos (n=5) and day 5/6 blastocysts (n=5) were used for microarray analysis, and three additional individual blastocysts (n=3) were used for RT-qPCR validation. Embryos were freshly collected (n=7) (i.e., embryos with inadequate quality for transfer or cryopreservation) or were from frozen samples (n=6) (couples without further parental projects). All couples gave their informed consent for embryos donation for research.

### 2.2. Mouse Samples

#### 2.2.1. Mice's Characteristics

Fertile B6CBA/F1 mice (aged 6-9 weeks, n=24) were obtained from Charles River (Saint-Aubin-Les-Elbeufs, France). Mice had water and food* ad libitum*. Females were superovulated with one i.p. injection of 10 IU pregnant mare serum gonadotrophin (Folligon; Intervet, Beaucouze, France) at 12:00, followed 48h later by one i.p. injection of 5 IU eCG (equine Chorionic Gonadotropin; Chorulon, Intervet, Beaucouze, France) to induce ovulation. Then, females were mated with fertile males. The presence of a vaginal plug on the following day indicated successful mating.

#### 2.2.2. Oocytes

24h after CG administration, females were killed by cervical dislocation. Oviducts were excised and flushed with M2 medium (Sigma Aldrich). MII oocytes were pooled (n=3 pools of 25, 40, and 40 oocytes, respectively), placed immediately in RLT RNA extraction buffer, and frozen at -80°C (Table 2).

#### 2.2.3. Day 1.5 Embryos and Day 3/4 Blastocysts

Pregnant mice were sacrificed on gestational day 1.5 or 3.5. Oviducts were excised and flushed with M2 medium (Sigma Aldrich). Morphologically normal day 1.5 embryos (2-cell) (n=3) and day 3.5 embryos (blastocysts) (n=7) were collected and placed individually in RLT RNA extraction buffer for storage at -80°C (Table 2). Morphologically normal day 1.5 embryos were defined as having an intact zona pellucida with equal or slightly different blastomeres in size, round, with no or up to 10% fragmentation with granules in cytoplasm. The grading of blastocysts as good morphological appearance was defined with equal size of blastomeres and presence of a small blastocele or presence of a large blastocele cavity and thin zona pellucida.

### 2.3. RNA Extraction

Total RNA was extracted using the RNeasy Micro Kit (Qiagen) for pooled human oocytes, and the PicoPure RNA Isolation Kit (Arcturus Bioscience) for human day 3 embryos and day 5/6 blastocysts as well as all mouse samples, according to the manufacturers' recommended protocols. RNA quantity and integrity were determined with an Agilent 2100 Bioanalyzer (Agilent Technologies, Palo Alto, CA). RNA samples processed for Affymetrix microarrays had RNA Integrity Number (RIN) values > 7.

### 2.4. Complementary RNA (cRNA) Preparation

Total RNA from MII oocytes (from 1 to 4.5 ng/*μ*l), EGA (day 1.5 mouse and day 3 human embryos; from 350 to 600 pg/*μ*l), and blastocysts (from 100 to 800 pg/*μ*l) underwent double rounds of linear amplification to generate suitable quantity of labelled cRNA, except for human day 3 embryos (three amplification rounds) [11]. 15 *μ*g of each amplified RNA sample was hybridized to Genome U133 Plus 2.0 arrays (Affymetrix, Santa Clara, CA, USA) or Mouse Genome 430 2.0 Arrays (Affymetrix).

### 2.5. Microarray Data Processing and Visualization

Scanned Gene Chip images were processed using the Affymetrix GCOS 1.4 software to obtain the intensity value signal and detection call for each probe set using the default analysis settings and global scaling as the first normalization method. Probe intensities were derived using the MAS5.0 algorithm. Checking normal distribution of microarray data was performed using the relative log expression plot. To compare the gene expression profiles between sample groups, a probe set selection using the absent/present ‘detection call' (present in at least 50% of samples) and a coefficient of variation (CV) ≥40% between samples was first performed. Then, the Significant Analysis of Microarrays (SAM, Stanford University [13]) was used to identify genes the expression of which varied significantly between MII oocytes (n=4) and day 3 embryos (n=5), MII oocytes and day 5/6 blastocysts (n=5), and day 3 embryos and day 5/6 blastocysts for human samples and between MII oocytes (n=3) and day 1.5 embryos (n=3), MII oocytes and day 3.5 blastocysts (n=3), and day 1.5 embryos and day 3.5 blastocysts for mouse samples (fold change, FC >2; false discovery rate, FDR < 5%). Then, significantly upregulated and downregulated genes were analysed using Ingenuity Pathway Analysis (IPA) software (Qiagen, Redwood City) to identify the top canonical pathways and regulator effect networks associated with each comparison. IPA predicted activation and inhibition of upstream and downstream regulators and utilized the regulator effects algorithm to connect identified upstream regulators with the dataset genes and downstream functions and diseases to generate a hypothesis with a consistency score >2 or <-2. Only consistent predicted relationships are shown in networks (i.e., inconsistent and not predicted interactions were deleted).

To obtain clear schemes of apoptotic and survival signalling during early human and mouse embryonic development, we performed a synthesis of all signalling pathways reached to the apoptosis and survival functions using IPA. This part was performed with the lists of upregulated genes obtained after SAM analyses as well as lists of genes that are not differentially expressed between all comparisons. To identify genes that were not differentially expressed between stages (MII* versus* EGA; MII* versus* blastocyst; EGA* versus* blastocyst), microarray data were first selected based on the ‘detection call' (present in at least 50% of samples) with a CV ≥10%. From these preselected lists, the genes identified by SAM were removed. Then, intersection of these lists of genes led to the identification of genes that were not differentially expressed between stages and between species.

### 2.6. Quantitative RT-PCR Analysis

Total RNA derived from single MII oocytes (human, n=4; mouse, n=3) and blastocysts (human day 5/6 blastocysts, n=3; mouse day 3.5, n=4) was used for reverse transcription (RT) in a final volume of 20 *μ*L with the SuperScript® First-Strand Synthesis System (Invitrogen), according to the manufacturer's instructions. Quantitative PCR (qPCR) was performed in 384-well plates (Sorenson Bioscience) on a Lightcycler® 480 Real-Time PCR System (Roche Diagnostics). cDNA was added to the Sybr Green PCR master mix (Roche Diagnostics) with 0.5*μ*M forward and reverse primers (primer sequences are in Supplementary Table S1) for amplification according to the following conditions: 10min at 95°C, then 50 cycles of 10s at 95°C, 20s at 63°C, and 25s at 72°C. At the end, a melting curve from 95°C to 63°C was performed to control primer specificity.* HPRT1* and* Lbr*, for human and mouse samples, respectively, were used as reference housekeeping genes because their mRNA expression level did not vary in the studied developmental stages. The relative expression ratios were calculated using the formula: E^ΔCt^_tested  primer_ / E^ΔCt^_HG_, where E is the qPCR efficiency and ΔCt = Ct control – Ct unknown. The E value was determined by a standard curve for each primer used (E = 10^−1/slope^).

### 2.7. Statistical Analysis

Data were compared with the Student's* t*-test and GraphPad Instat (GraphPad, San Diego, CA);* p*≤0.05 was considered to be statistically significant.

## 3. Results

### 3.1. Stage-Specific Human Gene Expression Profiles

**Figure 1:**
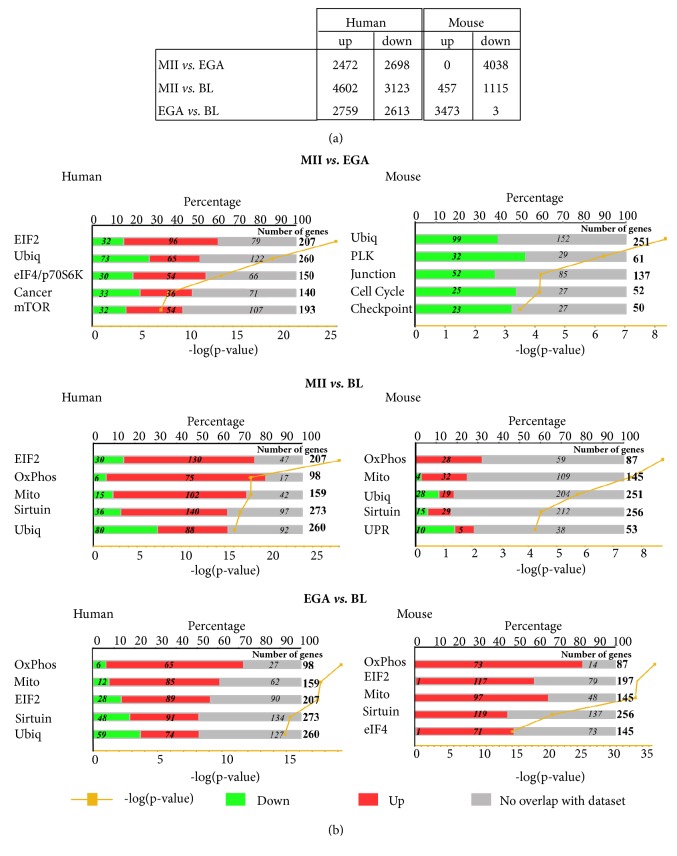
Number of genes that are differentially expressed during early human and mouse embryo development (a) and the top five canonical pathways associated with each stage in both species (b). Y-axis represents canonical pathways with stacked bar associated for each pathway with the number of downregulated (green color) and upregulated (red color) genes indicated in bold italic. The total number and percentage of downregulated and upregulated genes associated with each specific canonical pathway were indicated in bold to the right of each bar and to the top of the X-axis, respectively. At the bottom of the X-axis, the logarithmic p-value of each canonical pathway was indicated (orange). MII, metaphase II oocytes; EGA, embryonic genome activation stage; BL, blastocytes; EIF2, EIF2 signalling; Ubiq, protein ubiquitination pathway; eIF4/p70S6K, regulation of eIF4 and p70S6K signalling; Cancer, hereditary breast cancer signalling; mTOR, mTOR signalling; OxPhos, oxidative phosphorylation; Mito, mitochondrial dysfunction; Sirtuin, sirtuin signalling pathway; PLK, mitotic roles of polo-like kinase; Cell Cycle, cell cycle control of chromosomal replication; Junction, epithelial adherens junction signalling; Checkpoint, cell cycle: G2/M DNA damage checkpoint regulation; UPR, unfolded protein response.

A first selection of genes using a CV≥40% and a present detection call in at least 50% of the three comparison groups of human samples (i.e., MII oocytes* versus* day 3 embryos, MII oocytes* versus* day 5/6 blastocysts, and day 3 embryos* versus* day 5/6 blastocysts) identified 7618, 9660, and 8239 genes, respectively. Then, SAM analyses of these selected gene lists identified 5170, 7725, and 5372 genes that were differentially expressed in MII oocytes compared with day 3 embryos (EGA), MII oocytes compared with day 5/6 blastocysts, and day 3 embryos (EGA) compared with day 5/6 blastocysts, with a similar proportion of upregulated and downregulated genes in each comparison (Figure 1(a), Supplementary Table S2, S3, and S4). We then performed functional analyses with these three lists of genes to identify canonical pathways associated with each embryonic developmental stage. Indeed, the top five canonical pathways associated with the MII* versus* EGA stage were the EIF2 signalling (p=1.1E-26), the protein ubiquitination pathway (p=6.6E-20), the regulation of eIF4 and p70S6K signalling (p=2.1E-14), the hereditary breast cancer signalling (p=7.3E-9), and the mTOR signalling (p=4.9E-08) (Figure 1(b)). More precisely, numerous eukaryotic translation initiation factors were deregulated during the MII versus EGA stage including* EIF1* (x-2.4, FDR=0.006),* EIF5* (x25.5, FDR<0.0001),* EIF1AX* (x7.1, FDR=0.01),* EIF2S1* (-5.1, FDR<0.0001),* EIF2AK1* (x-12, FDR<0.0001),* EIF2AK2* (x-4.4, FDR=0.0002),* EIF2B1* (x-3.8, FDR=0.003),* EIF2B2* (x3.8, FDR=0.02),* EIF2S1* (x-2.9, FDR=0.0007),* EIF2S2* (x3.7, FDR<0.0001),* EIF2S3* (x2.1, FDR=0.04),* EIF3A* (x-5.4, FDR=0.001),* EIF3B* (x5, FDR=0.02),* EIF3D* (x7.2, FDR=0.02),* EIF3E* (x3.9, FDR=0.0004),* EIF3F* (x76.9, FDR=0.0009),* EIF3G* (x12, FDR<0.0001),* EIF3H* (x6.2, FDR<0.0001),* EIF3I *(x2.4, FDR=0.02),* EIF3J* (x25.3, FDR<0.0001),* EIF3K* (x3.5, FDR=0.003),* EIF3L* (x5.1, FDR<0.0001),* EIF3M* (x6.6, FDR<0.0001),* EIF4A3* (x24.9, FDR=0.009),* EIF4E* (x-3.4, FDR=0.001),* EIF4G1* (x-2.4, FDR=0.02),* EIF4G2* (x-2.1, FDR=0.004),* EIFG3* (x-2.66, FDR=0.003), and* EIF5B* (x2.8, FDR=0.005). Among the protein ubiquitination pathway, numerous ubiquitin specific peptidases coding genes were underexpressed in the EGA stage compared with the MII stage such as* USP1* (x-24.2, FDR=0.0003),* USP2* (x-2.4, FDR=0.02),* USP4* (x-3.1, FDR=0.0005),* USP10* (x-2.5, FDR=0.009),* USP11* (x-14, FDR=0.0004),* USP12 *(x-2.2, FDR=0.03),* USP14* (x-7.1, FDR=0.0002),* USP15* (x-4.4, FDR=0.006),* USP21* (x-3.7, FDR=0.001),* USP22* (x-3.1, FDR=0.005),* USP33* (x-2.2, FDR=0.02),* USP34* (x-3, FDR=0.0004),* USP35* (x-3.3, FDR=0.02),* USP37* (x-2.4, FDR=0.008),* USP44* (x-31.4, FDR<0.0001),* USP45* (x-9.7, FDR=0.02),* USP46* (x-5.1, FDR=0.009),* USP47* (x-2.9, FDR=0.009) excepted for* USP3* (x6.4, FDR=0.0005),* USP7* (x4.8, FDR<0.0001),* USP19 *(x4.1, FDR=0.02),* USP32* (x5.9, FDR<0.0001),* USP36* (x9.8, FDR=0.005),* USP38* (x6.6, FDR=0.006),* USP42* (x29.5, FDR<0.0001),* USP48* (x2.8, FDR=0.03), and* USP54* (x2.1, FDR=0.01). Underexpression of the majority of these peptidases was maintained or accentuated in the blastocyst (BL) stage compared with both MII and EGA stage.

On the other hand, several protein complexes of the proteasome coding genes were overexpressed in the EGA compared with the MII stage [as* PSMA5* (x3.3, FDR=0.01),* PSMA6* (x10.7, FDR<0.0001),* PSMB1* (x6.3, FDR<0.0001),* PSMB2* (x2.3, FDR=0.003),* PSMB3* (x2, FDR=0.002),* PSMB7* (x2.5, FDR=0.03),* PSMC1* (x3.1, FDR=0.02),* PSMC4* (x4, FDR=0.0007),* PSMC5* (x2.8, FDR=0.002),* PSMC6* (x2.2, FDR=0.04),* PSMD4* (x7.2, FDR=0.0007),* PSMD8* (x14.5, FDR=0.005),* PSMD12* (x5.6, FDR=0.003),* PSMD13* (x8.1, FDR=0.001),* PSMD14* (x19.8, FDR=0.002), and* PSME2* (x18.2, FDR=0.0002)]. Except for the* PSMA5*, all of them were also overexpressed at the BL compared with both MII and EGA stage. Moreover, other members of the proteasome complex were overexpressed in the BL compared with the two other stages including* PSMB4*,* PSMB5*,* PSMB6*,* PSMC2*,* PSMD6,* and* PSMD7*.

In addition to several members of the eIF4 group, numerous gene encoding ribosomal proteins (RP) reached to the regulation of eIF4 and p70S6K signalling were overexpressed in the EGA compared with the MII stage including* RPS7*,* RPS9*,* RPS10*,* RPS11*,* RPS12*,* RPS13*,* RPS14*,* RPS16, RPS17*,* RPS18*,* RPS20*,* RPS21*,* RPS23*,* RPS28*,* RPS29*,* RPS15A*,* RPS27A*,* RPS27L*,* RPS3A*,* RPS4X,* and* RPSA.* All these genes were also overexpressed in the BL group compared with MII stage (Figure 1(b)).

Among the top five canonical pathways associated with the MII* versus* BL stage or EGA versus BL stage, the oxidative phosphorylation, the mitochondrial dysfunction, and the sirtuin signalling pathway were predominant pathways associated with the BL stage. Majority of genes related to these canonical pathways were overexpressed in the BL compared with both MII and EGA stage (Figure 1(b)).

**Figure 2:**
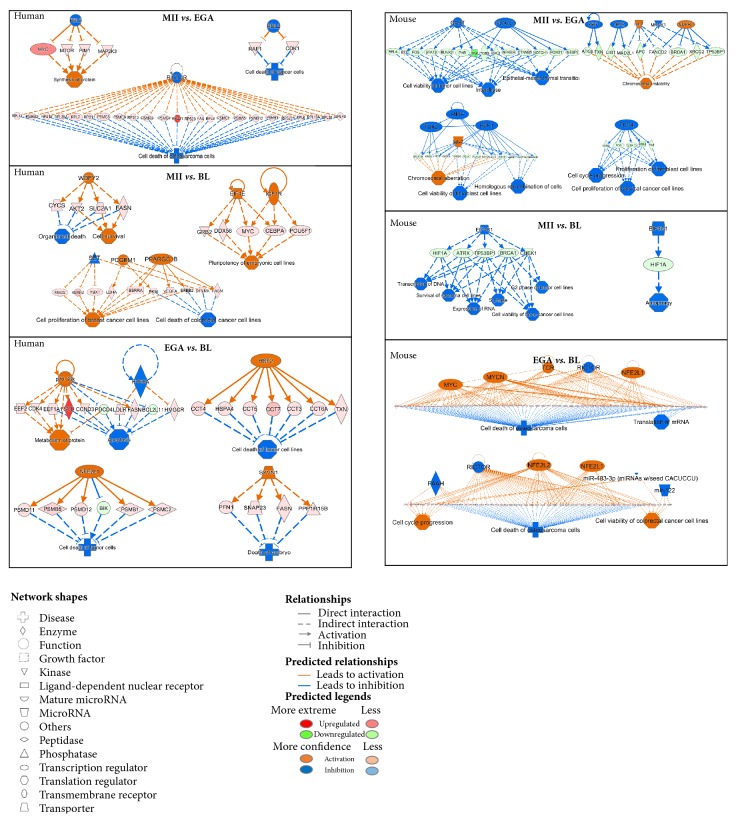
The predicted top regulator effects networks in the MII* versus* EGA, MII* versus* BL, and EGA* versus* BL stages in human (left) and mouse (right). Upstream regulators are displayed in the top tier while diseases and functions are displayed in the bottom. The dataset genes that connect the upper regulator to the lower diseases and functions are in the middle tier and causal relationships are displayed with blue (inhibition) and orange (activation) line. Significant upregulated and downregulated genes from the dataset genes are in red and green, respectively. The color intensities of the connected genes were proportional to their fold changes.

Based on significant upregulated and downregulated genes, ingenuity pathways analysis predicted inhibition of upstream regulators such as RICTOR (x-15.4, FDR<0.0001) and RBL2 (x-7.2, FDR<0.0001) at the EGA compared with the MII stage with a consistency score of -6.8 and -3.7, respectively (Figure 2). The RICTOR upstream regulator targeted a number of genes including several gene encoding ribosomal proteins such as* RPL11* (x2.3, FDR=0.009),* RPL35A* (x8.5, FDR<0.0001),* RPS11* (x7.5, FDR<0.0001),* RPS13* (x2.5, FDR=0.0005),* RPS18* (x8.5, FDR<0.0001), and* RPS21* (x245, FDR=0.003) that in turn inhibited indirectly cell death of day 3 human embryos. On the other hand, the underexpression of the RBL2 upstream regulator predicted to inhibit cell death via the protooncogene RAF1 and the cyclin dependent kinase CDK1 and to activate protein synthesis by targeting the protooncogenes MYC and PIM1 as well as mTOR and MAP2K3 kinases.

At the BL compared with the MII stage, activation of WDFY2 and PPARGC1B upstream regulator, with a consistency score of 4.5 and 3.8, respectively, predicted to inhibit cell death and promote cell survival and cell proliferation by targeting numerous genes including* FASN* (x3.6, FDR=0.0006),* AKT2* (x2.5, FDR=0.0002), and* VEGFA* (x12.4, FDR=0.003). Compared with the EGA stage, predicted activation of RPS6KB1 (consistency score of of 4.4), HSF2 (consistency score of 2.3), NFE2L1 (consistency score of 2.3), and SYVN1 (consistency score of 2) upstream regulators stage leads to the inhibition of cell death and apoptosis of human blastocysts via the downregulation of proapoptotic members of the BCL2 family such as* BCL2L11* (x-5.9, FDR=0.0005), and* BIK* (x-7.5, FDR=0.006) and the upregulation of transcripts from the proteasomes including* PSMB1* (x3.3, FDR<0.0001),* PSMB5* (x12, FDR<0.0001),* PSMD11* (x4.9, FDR=0.0003),* PSMD12* (x6.2, FDR=0.0005), and* PSMC2* (x4.9, FDR=0.0002) (Figure 2).

### 3.2. Stage-Specific Mouse Gene Expression Profiles

Using the same selection criteria for the mouse microarray data analysis, 13937, 8350, and 9480 genes were identified by comparing mouse MII oocytes and day 1.5 embryos, MII oocytes and day 3.5 blastocysts, day 1.5 embryos, and day 3.5 blastocysts, respectively. SAM analyses identified 4038, 1572, and 3476 genes that were differentially expressed in each comparison (Figure 1(a); Supplementary Table S5, S6, and S7). All genes were downregulated at the EGA stage (day 1.5 embryos) compared with MII oocytes, while most genes (99.9%) were upregulated in blastocysts compared with day 1.5 embryos (EGA) (Figure 1(a)).

From the MII* versus* EGA stage comparison, the top five canonical pathways were the protein ubiquitination pathway (p=4.5E-09), the mitotic roles of polo-like kinase (p=6.2E-07), the epithelial adherens junction signalling (p=6.6E-05), the cell cycle control of chromosomal replication (p=7.0E-05), and the G2/M DNA damage checkpoint regulation (p=3.1E-04). All genes related to these signalling pathways were underexpressed in the EGA compared with the MII stage. The oxidative phosphorylation, mitochondrial dysfunction, and the sirtuin signalling pathway were the top canonical pathways associated with the BL compared with both MII and EGA stage (Figure 1(b)).

At the EGA compared with the MII stage, IPA predicted inhibition of upstream regulators such as* Gast*/*Foxc1*,* Tbx2*/*Mitf*/*E2f1*,* Tcf4*,* Xbp1*/*Tbx2*/*Mknk1* with high consistency scores between 4 to 6.5. Underexpression of Gast (x-5.1, FDR<0.0001) and* Foxc1* (x-2.2, FDR=0.02) transcripts promotes cell cycle interphase and cell viability. These two upstream regulators target numerous underexpressed genes including* Stat3* (x-3.8, FDR=0.01),* Itgb3* (x-3.1, FDR=0.001), and* Jag1* (x-44.7, FDR<0.0001). In the same way, the underexpression of* Tbx2*,* Mitf*, and* E2f1* regulators activate the cell viability and promote homologous recombination of cells. Also, the inhibition of the* Tcf4* regulator promotes cell cycle progression and cell proliferation.

At the BL compared with MII stage, inhibition of the* Eif4g1* predicted to promote the transcription of DNA, expression of RNA, cell viability, and survival with a consistency score of 9.3. However, compared with the EGA stage, IPA predicted inhibition of upstream regulators such as* Rictor* and* Faah* and activation of* Myc*,* Mycn*,* Tcr, Nfe2l1, *and* Nfe2l2 *leading to the inhibition of cell death and activation of the cell cycle progression and cell viability in mouse blastocysts (Figure 2).

### 3.3. Stage-Specific Human Survival-Related Gene Expression Profiles

**Figure 3:**
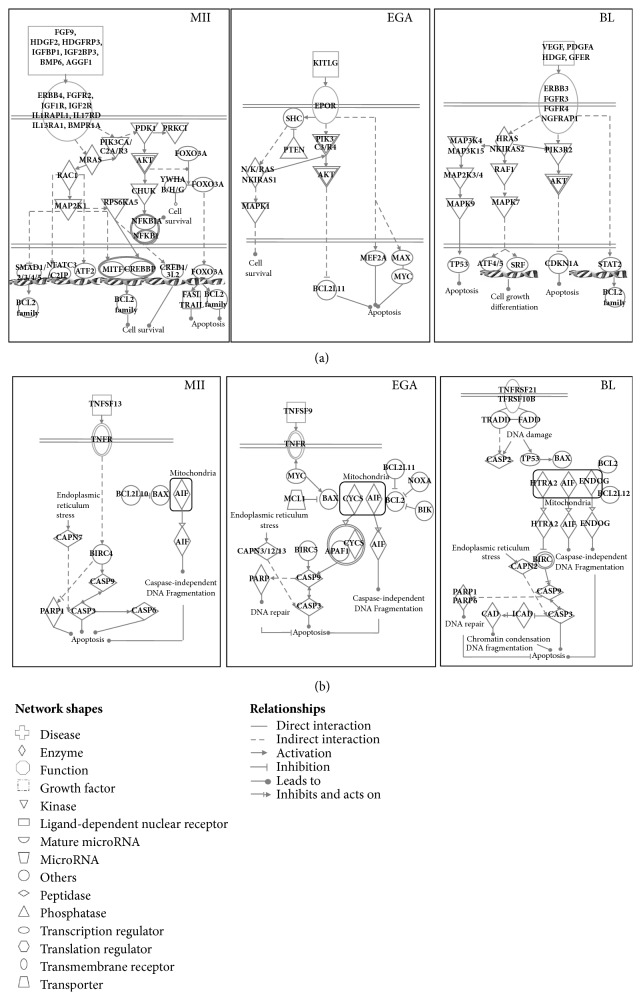
Survival (a) and apoptotic pathways (b) during human early embryonic development. The IPA software was used with lists of genes identified as not differentially expressed as well as with upregulated genes issued from each comparison to identify and synthesis survival and apoptotic pathways. MII, metaphase II oocytes; EGA, embryonic genome activation stage; BL, blastocytes.

Among the differentially expressed genes (Figure 1(a)), many genes encoding components of major survival signalling pathways were expressed during early embryonic development, including growth factors and cytokines and their receptors, factors and second messengers of the PIK3 and MAPK pathways and downstream transcription factors. In human samples, survival-related genes were highly represented in MII oocyte samples. Indeed, genes encoding several growth factors (such as* HDGFL2, HDGFL3, FGF9, IGFBP1, IGF2BP3, AGGF1, * and* BMP6*), growth factor receptors (*ERBB4, FGFR2, FGFR1OP, IGF1R, IGF2R, IL1RAPL1, IL17RD, IL13RA1, *and* BMPR1A*), intracellular mediators of the MAPK (*PDK1, PRKCI, RAC1, *and* MAP2K1*) and PI3K pathways (*PIK3CA, PI3KC2A, *and* PIK3R3*), and downstream transcription factors (*SMAD1, SMAD2, SMAD3, SMAD4, SMAD5, NFATC3, NFATC2IP, ATF2, MITF, CREBBP, CREB1, CREB3L2, *and* FOXO3A*) were overexpressed at this stage compared with day 3 embryos and day 5/6 blastocysts (Figure 3(a)). In day 3 embryos, the genes encoding KIT ligand (*KITLG*), erythropoietin receptor (*EPOR*), components of the PIK3/AKT (*PIK3C3, PIK3R4, *and* PTEN*) and MAPK (*NRAS, KRAS, NKIRAS1, *and* MAPK1*) pathways, and the transcription factors* MYC*,* MAX,* and* MEF2A* were overexpressed compared with MII oocytes and day 5/6 blastocysts (Figure 3(a)). Finally, several genes encoding growth factors (*VEGFA, PDGFA, HDGF, *and* GFER*) and their receptors (*FGFR3, FGFR4, ERBB3, *and* BEX3*) were specifically overexpressed in day 5/6 blastocysts (Figure 3(a)). Moreover, the genes encoding PIK3R2, several factors involved in the MAPK pathway (*HRAS, NKIRAS2, RAF1, MAPK7, MAPK9, MAP3K4, MAP3K15, MAP2K3, *and* MAP2K4*), and transcription factors, such as* TP53*,* ATF4*,* ATF5, SRF,* and* STAT2,* were upregulated at this stage.

### 3.4. Stage-Specific Mouse Survival-Related Gene Expression Profiles

**Figure 4:**
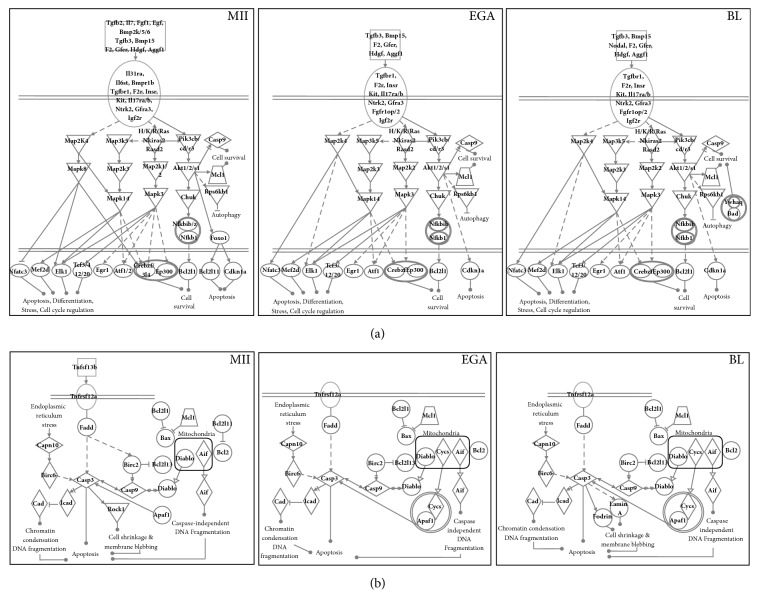
Survival (a) and apoptotic pathways (b) during mouse early embryonic development. The IPA software was used with lists of genes identified as not differentially expressed as well as with upregulated genes issued from each comparison to identify and synthesis survival and apoptotic pathways. MII, metaphase II oocytes; EGA, embryonic genome activation stage; BL, blastocytes (legends are in Figure 3).

Conversely, in mouse preimplantation embryos, most of the genes encoding survival-related factors were similarly expressed at all the stages under study (Figure 4(a)). Indeed, several genes encoding growth factors/cytokines (*Tgfb3, F2, Gfer, Hdgf, *and* Aggf1*) and their receptors (*Tgfbr1, F2r, *and* Ntrk2*), intracellular actors of the PIK3/AKT (*Pik3cb, Pik3cd, Pik3r3, Akt1, *and* Akt2*) and MAPK (*Hras, Kras, Rras, Mapk3, Map2k2, Map2k3, Map2k4, *and* Map3k5*) pathways, and several transcription factors (*Nfatc3, Mef2d, Tcf3, Atf1, *and* Crebzf*) were similarly expressed in MII oocytes, day 1.5 embryos, and day 3.5 blastocysts. Nevertheless, a number of genes were specifically overexpressed in mouse MII oocytes, such as genes encoding growth factors and cytokines (*Fgf1, Egf, Igf2bp3, Tgfb2, Bmp2k, Bmp5, Bmp6,* and* Il7*), receptors (*Bmpr1b, Il6st, *and* Il31ra*),* Mapk8*,* Map2k1*, and the transcription factors* Foxo1, Tcf4, Atf2,* and* Creb3l4* (Figure 4(a)). Moreover,* Igf2bp3, Bmp6, Map2k1, and Atf2* were upregulated in both human and mouse MII oocytes (Figures 3(a) and 4(a)).

### 3.5. Stage-Specific Human Apoptosis-Related Gene Expression Profiles

Several components of the apoptotic machinery were also expressed during early embryonic development. In human samples,* TNFSF13* (tumour necrosis factor ligand),* BCL2L10*,* CASP6,* and* XIAP* were overexpressed in MII oocytes compared with day 3 embryos and day 5/6 blastocysts (Figure 3(b)). In day 3 embryos, other apoptosis-related genes were specifically upregulated, including* TNFSF9*, the BCL2 family members* MCL1, BCL2L11, PMAIP1* and* BIK, BIRC5,* and* CYCS* (Figure 3(b)). At the blastocyst stage,* TNFRSF21, TNFRSF10B, TRADD, FADD, BCL2L12, CASP2, HTRA2, ENDOG, DFFB, DFFA,* and* PARP6* were overexpressed (Figure 3(b)). Conversely,* CASP3, CASP9, AIF,* and* PARP1* were expressed at all stages under study.

### 3.6. Stage-Specific Mouse Apoptosis-Related Gene Expression Profiles

In mouse samples, most apoptosis-related genes (*Tnfrsf12a, Fadd, Bcl2l1, Mcl1, Birc2, Birc6, Casp3, Casp9, Diablo, Aif, Apaf1, *and* Dffa*) were expressed at all studied stages (MII oocytes, day 1.5 embryos, and day 3.5 blastocysts) (Figure 4(b)). Conversely,* Tnfsf13b, Bcl2l11,* and* Bcl2l13* were overexpressed in MII oocytes (Figure 4(b)).

*CASP3*,* CASP9,* and* AIF* were expressed throughout early mouse and human embryonic development.

### 3.7. Validation of Gene Expression

**Figure 5:**
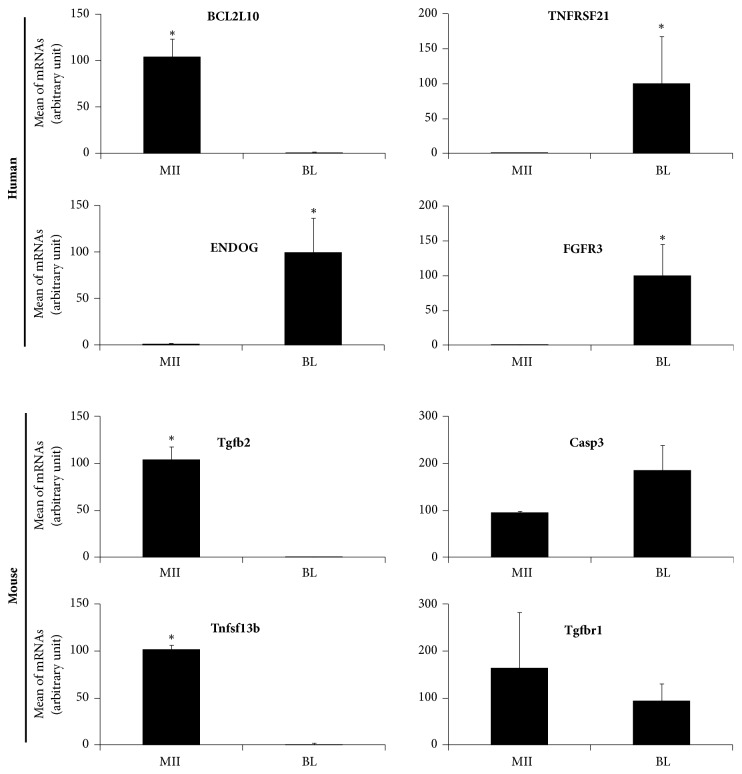
RT-qPCR validation of some the microarray gene expression data. Bars represent the mean ± SEM. MII: MII oocytes, BL: blastocysts. *∗* p<0,05.

Four differentially expressed human genes implicated in the survival and apoptotic pathways (*BCL2L10*,* TNFRSF21*,* ENDOG,* and* FGFR3*) were selected, on the basis of their fold change, for validation. Analysis of the RT-qPCR data confirmed the increased expression of* BCL2L10* in human MII oocytes in comparison with blastocysts, and the higher* TNFRSF21*,* ENDOG,* and* FGFR3* expression in human blastocysts than in MII oocytes (Figure 5).

Analysis of the RT-qPCR data confirmed that* Tgfb2* and* Tnfsf13b*, but not* Tgfbr1 *and* Casp3*, were overexpressed in mouse MII oocytes compared with blastocysts (Figure 5).

## 4. Discussion

As the first stages of embryogenesis are characterized by rapid cell proliferation, an optimal balance between survival and apoptotic signals must be maintained. Here, we have analysed the global gene expression profiles and compared the top canonical pathways and top regulators effect networks during early mouse and human embryonic development.

During early embryonic development, the maternal-to-zygotic transition is a complex process that can be subdivided into two interrelated processes: first, a subset of maternal mRNAs and proteins is eliminated; second, zygotic transcription is initiated. The timing and scale of these two events differ across species. In the mouse, zygotic gene activation involved two major transient waves of de novo transcription. The first wave corresponds to zygotic genome activation peaks at the 2-cell to 4-cell embryo stages; the second wave, named mid-preimplantation gene activation precedes the dynamic morphological and functional changes from the morula to blastocyst stage [14]. According to our mouse microarray data reported, at the 2-cell stage, the transcript levels of maternally stored RNAs is higher than those of embryonic genome. This finding is consistent with previous reports showing a minor wave of genome activation at the early 2-cell stages [15, 16]. Consequently, as we are probably not at the optimal time to visualize the peak of the zygotic genome activation characterized in the present study by the absence of upregulated genes in the MII versus EGA comparison, this circumstance affects also the difference in gene expression profiles between EGA and BL stage. In human, the EGA occurs between the 4- to 8-cell and the difference in gene expression profiles according to the embryonic stage reported in the present study were consistent with others [17, 18]. In addition, the EGA is confirmed by the activation of the EIF2 signalling and the implication of eIF4/p70S6K signalling that play crucial roles in the initiation and regulation of the translation, as judged by the number of genes encoding ribosomal proteins and translation initiation factors that were overexpressed in human day 3 embryos. Moreover, the canonical pathway analysis showed the involvement of genes reached to the protein ubiquitination pathway, characterized by the underexpression of numerous genes encoding ubiquitin specific peptidases and the overexpression of proteasome genes indispensable for the elimination of maternal proteins. On the other hand, the resulting regulator effects networks revealed the activation or inhibition of regulators targeting functions articulated around the cell death, cell viability and survival. Therefore, we focused our analyses on the expression of major components of the apoptotic and survival pathways during early mouse and human embryonic development.

It is well argued that oocytes and early embryos are highly sensitive to diverse exogenous factors such as temperature, oxygen, pH, nutrient restriction, and stress, particularly under IVF/ICSI procedure. Many of these factors can affect negatively the endoplasmic reticulum (ER) and protein synthesis inducing ER stress and unfolded/misfolded protein responses for which oocytes/embryos will have to face to survive [19]. Indeed, accumulation of unfolded/misfolded protein in the ER induced ER stress-mediated apoptosis especially via the eIF2 signalling pathway. As reported here, the eIF2 signalling pathway was predominant during all stages of human early embryos cultured* in vitro* and was restricted to the blastocyst stage (*in vivo*) in mouse, reinforcing the notion that apoptosis was more susceptible under* in vitro* maturation conditions. Expression of EIF2S1 mRNA was expressed throughout human embryo development* in vitro* with a stronger expression at the MII oocyte stage compared with both EGA and blastocyst stage. Interestingly, predicted activation of SYVN1 upstream regulator at the blastocyst stage targeted apoptosis inhibition via the downregulation of proapoptotic factors including BCL2L11 and BIK. In addition, SYVN1 (synoviolin 1), encoding a protein involved in ER-associated degradation, has been previously reported to promote EIF2S1 ubiquitylation and degradation, thereby preventing apoptosis in renal tubular epithelial cells [20]. Indeed, SYVN1 could provide a protective mechanism by suppressing apoptosis in human blastocyst via the EIF2S1 ubiquitylation and degradation. This finding is also consistent with the overexpression of several proteasome genes at the human blastocyst stage. In addition, SYVN1-deficient mice died in utero around embryonic day 13.5, demonstrating its indispensable role in maintenance of life [21].

On the other hand, predicted activation of NFE2L1 (nuclear factor, erythroid 2 like 1) upstream regulator at the blastocyst stage in both human and mouse provided also a protective mechanism against distinct cellular stressors, particularly those derived from the ER inducing ER stress-mediated apoptosis. More precisely, NFE2L1 regulated expression of the 26S proteasomal subunits as an adaptive recovery response to inhibition of the proteasome [22]. In the same way, activation of the heat shock transcription factor 2 (HSF2) upstream regulator provided another possible protective mechanism against cell death in human at the blastocyst stage. In the mouse,* HSF2* was absent at the zygote stage and starts to be expressed at the 4- to 8-cell embryos stages with a more detectable level at the blastocyst stage [23]. In our study,* HSF2* was expressed in 50% of the human unfertilized MII oocytes, all blastocyst and absent at the EGA stage, suggesting a similar profile of* HSF2* expression throughout early embryo development between human and mouse. Although Hsf2 knockout mice were viable and fertile, HSF2 is required for heat shock protein expression (heat shock response) inducing a protective response and, thereafter, cell survival [23]. Indeed, HSF2 deficient blastocyst displayed higher sensitivity to cell death under stress conditions.

Early developmental programming processes required ribosome biogenesis which is essential for protein synthesis, cell growth and proliferation. Predicted activation of the ribosomal protein S6 kinase B1 (RPS6KB1, also called P70S6K) upstream regulator at the blastocyst stage in human leads to the activation of the protein metabolism. RPS6KB1 phosphorylates the 40 S ribosomal protein S6 in response to mitogen allowing the upregulation of mRNA translation. The role of RPS6KB1 as an important downstream targets of mTOR and PI3K in the growth control of cell and organism has been intensively documented [24].

As potential mitogenic factors involved in human blastocysts, several growth factors such as VEGFA, PDGFA, HDGF, and GFER that are specifically overexpressed at the blastocyst stage appeared as good potential candidates. Conversely, mouse blastocysts did not show a specific expression profile. The angiogenic factor VEGFA is an important regulator of embryo implantation. For instance,* in vivo* VEGFA neutralization using a specific antibody leads to inhibition of blastocyst implantation in rhesus monkey [25]. In humans, VEGFA expression level is higher in uterine fluid samples from fertile than infertile women during the implantation window [26]. Furthermore, addition of human recombinant VEGFA to culture medium improves mouse blastocyst outgrowth and significantly increases human endometrial cell adhesion [26]. These data suggest that VEGFA has autocrine and paracrine actions during embryo development and that it promotes angiogenesis at the implantation site. PDGFA is overexpressed in human trophectoderm cells and expression of its receptor PDGFRA is increased in receptive endometrial cells during the implantation window [27]. Addition of PDGF to* in vitro* cultured mouse blastocysts promotes trophoblast outgrowth, suggesting a role in implantation [27, 28]. PDGFA signalling is also involved in the development and survival of inner cell mass-derived primitive endoderm cells [29, 30]. ERBB3 is a member of the epidermal growth factor receptor (EGFR) family that also could play an important role during embryo implantation [31]. Moreover, GFER, FGFR3, and FGFR4 were previously reported to be specifically upregulated in human trophectoderm cells [27, 32].

During human embryonic genome activation, canonical pathway analyses showed the downregulation* RICTOR (*RPTOR independent companion of MTOR complex 2) and* RBL2 (*RB transcriptional corepressor like 2) gene as relevant candidates as upstream regulators for inhibition of apoptotic machinery and activation of the protein synthesis in humans day 3 embryos. According to our data,* Rictor* was downregulated specifically at the blastocyst stage in mouse. RICTOR and mTOR are components of a protein complex that integrates nutrient- and growth factor-derived signals to regulate cell growth. Rictor-null mice and a mutant mouse model with oocyte-specific deletion of* Rictor *showed impairment of both folliculogenesis and embryonic development [33, 34]. More precisely, rictor-null mice exhibited placental defects and embryonic lethality at E11.5 while conditional knockout mice exhibited progressive loss of follicles and fertility due to excessive follicular atresia and became infertile at the age of 8 months, demonstrating that Rictor/mTOR functions in oocytes to protect follicles from apoptosis. The signalling pathway identified in follicle survival is mediated by the Rictor/mTOR/Akt axis that in turn inhibits Foxo3 transcription factor inducing a decrease in the protein levels of several proapoptotic factors such as Bad, Bax. However, as the conditional mouse model phenotype develops normally, this finding suggested that Rictor was not necessary for embryonic genome activation or that sufficient quantity of maternal transcripts controlling the Rictor signalling were present to support preimplantation embryonic development. Accordingly, our data suggested that RICTOR was also dispensable for the human zygotic genome activation.

Components of the apoptotic machinery were overexpressed during early mouse and human embryonic development.* TNFSF13* and* Tnfsf13b* (members of the TNF ligand family) were specifically overexpressed in human and mouse MII oocytes. TNFSF13 and TNFSF13B show the highest sequence identity within the TNF family, and both can bind to the TNFRSF13 and TNFRSF13B receptors [35]. TNFSF13 has an antiapoptotic effect in death ligand-induced apoptosis in a glioma cell line and has been associated with enhanced levels of XIAP, a member of the inhibitor of apoptosis (IAP) family that prevents cell death by directly inhibiting caspase activity [36].* XIAP* was specifically upregulated in human MII oocytes.* TNFSF13 *overexpression in human oocytes was reported previously [17, 37], and it can have both proapoptotic and antiapoptotic functions, according to the used cell line [38, 39]. TNFSF13B is involved in the regulation of B cell survival [40]. This effect is associated with upregulation of antiapoptotic genes of the BCL2 family, such as* BCL2* and* BCL2L1*, and degradation of the proapoptotic protein BCL2L11. Our finding that* Bcl2l11* is overexpressed in mouse MII oocytes suggests that* Tnfsf13b* expression might be important for BCL2L11 downregulation, thus promoting oocyte survival.* BCL2L10 *was overexpressed in human MII oocytes, as previously shown [17, 18, 41–43]. It promotes survival by inhibiting the mitochondrial release of cytochrome c, a key caspase 3 activator [44]. Furthermore, the localization of this antiapoptotic protein (i.e., mitochondria or nucleus) has been linked to human embryo quality up to the blastocyst stage [45]. The* CASP6* gene, which encodes the caspase 6 effector, also was overexpressed in human MII oocytes, as previously described [17], and could play a role downstream of the TNF pathway via TNFR1.

*TNFSF9*, which encodes another TNF ligand family member, was overexpressed in human day 3 embryos. This cytokine has several roles in immune cells, including proliferation, differentiation, apoptosis and survival [46]; however, its function in preimplantation embryos is not known. Several members of the BCL2 family, including the antiapoptotic* MCL1* and the proapoptotic* BCL2L11*,* BIK,* and* PMAIP1*, were upregulated at this stage, in agreement with our previous results [47]. Expression of* BIK*, which encodes a BCL2-interacting protein, was increased in day 3 human embryos, confirming previous transcriptomic studies [17, 48] and our published data [47]. This cell death factor could be implicated in the release of cytochrome c from mitochondria in stress conditions [49].* CYCS*, the gene encoding cytochrome c, also was upregulated in day 3 human embryo, as reported previously [17]. Moreover, mitochondrial apoptosis can be regulated via p53 and members of the BIRC family (e.g., BIRC6 that is essential for mouse embryonic development) [50].* BIRC5*, another member of this proteins family that is expressed during early embryonic development in humans and other mammalian species, was increased at this specific stage. In addition, BIRC5 expression inhibition promotes caspase-dependent apoptosis, and TGF*α* inhibits apoptosis through BIRC5 upregulation in mouse preimplantation embryos [51, 52], suggesting a protective role.

*ATF4 *expression also progressively increased in early embryos and blastocysts, as previously reported in day 3 human embryos using single-cell RNA-Seq [17, 48, 53]. Interestingly, this transcription factor promotes the expression of the BH3-only protein PMAIP1 [54]. In agreement,* NOXA* was upregulated in day 3 embryos. The canonical pathway analysis showed the downregulation* RICTOR* gene as relevant candidate as upstream regulator for inhibition of apoptotic machinery in humans day 3 embryos. This finding is consistent with the mouse model of* Rictor* invalidation showing impairment of both folliculogenesis and embryonic development [33, 34].

*TNFRSF21* and* TNFRSF10B* (encoding two death receptors of the TNFR family) and* TRADD* and* FADD*, which encode their adaptor proteins, were overexpressed in human blastocysts.* TNFRSF21* function during preimplantation development is not known but its overexpression is correlated with the decline of oocyte competence with ageing [43]. Conversely,* TNFRSF10B* is expressed in human blastocysts [55] and induces apoptosis in mouse blastocysts [56].* TP53* also was upregulated at this stage.* TP53 *encodes a tumour suppressor that maintains genomic stability in response to internal and external stress signals through the regulation of several processes, including apoptosis induction. Cell fragmentation and abnormal embryonic development are associated with increased* TP53 *levels in human embryos [57, 58]. Interestingly,* in vitro* fertilization and embryo culture increase* p53* expression in mouse blastocysts [57]. In addition, elevated Tp53 expression following culture results in reduced embryonic viability after mouse embryo transfer [59], and* Tp53 *genetic ablation improves mouse embryo development* in vitro* [60]. Recent data have shown that the BCL2 family member BCL2L12 can interact with and inhibit p53-mediated apoptosis induced by DNA damage and senescence in somatic cells [61]. It is tempting to speculate that* BCL2L12 *upregulation in human blastocysts could represent a way to regulate p53 apoptotic action. However, in mouse day 1.5 embryos and blastocysts, apoptotic genes were not significantly overexpressed.

In the present study, we point major differences in gene expression profiles between human and mouse samples. Although many fundamental aspects of the early stages of embryonic development in humans are found to be conserved in other mammals, recent advances in gene editing such as CRISPR-Cas9 system facilitating functional studies of specific genes highlighted and confirmed major differences during early embryo development between human and mouse [62–66]. Now, it is well recognised that the bovine model was greater similar to human than the mouse for study human early embryo development, and represent a model more accessible than monkeys [65, 67, 68]. As example, OCT4 is required for maintaining NANOG-positive epiblast cells in the inner cell mass of bovine blastocysts, similarly to human embryos but in contract to the mouse development [65]. However, for several aspects of the early embryo development, the mouse model can provided relevant information for the understanding of human early embryo development [66, 69].

Several limiting issues of our present study must be addressed. First, maturation conditions were not comparable between species (*in vivo* versus* in vitro*). For example, it was previously reported that maternal-derived genes are aberrantly expressed in mouse oocytes cultured* in vitro* compared with oocytes that developed* in vivo*, thus affecting the oocyte developmental capacity, and subsequently embryo viability [70–72]. In addition, various differences (morphology, cell number and allocation, apoptosis rate, and mRNA expression profiles) have been observed between embryos derived* in vitro* and* in vivo* [73–76], while some other studies reported a modest or absence of the impact of* in vivo* versus* in vitro* maturation conditions on gene expression profiles [70, 77]. In addition, human embryos were produced using the ICSI procedure while mouse embryos were collected from animals. Second, according to the French health authorities, biomedicine agency, only human unfertilized MII oocytes (aged human oocytes) as well as embryos with, for the majority, inadequate quality for transfer or cryopreservation have been analysed by DNA microarray and compared to the* in vivo* mouse model. During fertilization, the sperm transmits not only nuclear DNA but also coding (mRNAs) and noncoding RNAs (miRNAs, piRNA, siRNA) that could influence and regulate maternal messengers, and subsequent early embryonic development. Although coding and noncoding RNAs from paternal origin were expressed in fertilized MII oocytes, the real impact on fertilization and subsequent early preimplantation development is still uncertain. While a limited role of sperm-borne prototypical miRNAs in mammalian fertilization or early preimplantation development has been previously reported because their concentrations would be too low to have significant effects [78], other recent articles demonstrated the key roles of paternally derived miRNAs in embryonic development [79, 80]. In addition, the aneuploidy status of human preimplantation embryos has not been evaluated due to the French health authorities which does not authorize the preimplantation genetic diagnostic excepted in the executive for family genetic disease, strong probability, particular gravity, or incurability. Lastly, as only RNA samples from human day-3 embryos underwent triple amplifications to generate sufficient material for Affymetrix microarrays analyses, we cannot exclude the possibility that there is a potential bias on gene expression profiles between samples having undergone double versus triple rounds of linear amplification.

In conclusion, in the present study we show that components of the apoptotic and survival pathways are expressed during early mouse (*in vivo*) and human (*in vitro*) embryonic development. However, their expression profile is quite different between species as expected. Nevertheless, our study shows that while most of the genes associated with survival and death are expressed throughout early mouse embryonic development* in vivo*, their expression is more stage-specific in human samples (*in vitro* conditions). The potential roles of some of these genes as candidates for human oocyte/embryo quality assessments need to be investigated.

## Figures and Tables

**Table 1:** Number of human samples and patients for DNA microarray and RT-qPCR experiments. The number of fresh and cryopreserved embryos used were indicated in brackets.

	DNA microarray experiments		RT-qPCR experiments	
	Number of samples (fresh, cryopreserved)	Number of patients	Number of samples (fresh, cryopreserved)	Number of patients
Unfertilized MII oocytes	4*∗*	39	4	2
Day 3 cleavage embryos	5 (3, 2)	2	0	0
Day 5/6 blastocysts	5 (3, 2)	2	3 (1, 2)	2

*∗*, pools.

**Table 2:** Number of mice samples and females used for DNA microarray and RT-qPCR experiments.

	DNA microarray experiments		RT-qPCR experiments	
	Number of samples	Number of mice	Number of samples	Number of mice
Unfertilized MII oocytes	3*∗*	11	3	1
Day 1.5 embryos	3	6	0	0
Day 3/4 blastocysts	3	5	4	1

*∗*, pools.

## Data Availability

Numerous additional supplementary tables including the majority of our results were included in the present manuscript.

## Supplementary Materials

Supplementary Materials Supplementary Table S1: sequence of the forward and reverse primers used for the RT-qPCR analysis. Supplementary Table S2: the 200 most upregulated and downregulated in the EGA compared with the MII stage in human. MII, metaphase II oocytes; EGA, embryonic genome activation stage. Supplementary Table S3: the 200 most upregulated and downregulated in the BL compared with the MII stage in human. MII, metaphase II oocytes; BL, blastocysts. Supplementary Table S4: the 200 most upregulated and downregulated in the BL compared with the EGA stage in human. EGA, embryonic genome activation stage; BL, blastocysts. Supplementary Table S5: the 200 most downregulated in the EGA compared with the MII stage in mouse. MII, metaphase II oocytes; EGA, embryonic genome activation stage. Supplementary Table S6: the 200 most upregulated and downregulated in the BL compared with the MII stage in mouse. MII, metaphase II oocytes; BL, blastocyte. Supplementary Table S7: the 200 most upregulated and the 3 downregulated in the BL compared with the EGA stage in mouse. EGA, embryonic genome activation stage; BL, blastocyte.
