# Supplementary material for: Global, Survival, and Apoptotic Transcriptome during Mouse and Human Early Embryonic Development
Source: Biomed Res Int. 2018 Nov 1;2018:5895628. doi: 10.1155/2018/5895628 (PMC6236930; doi:10.1155/2018/5895628)
Supplement: Supplementary Materials — Supplementary Table S1: sequence of the forward and reverse primers used for the RT-qPCR analysis. Supplementary Table S2: the 200 most upregulated and downregulated in the EGA compared with the MII stage in human. MII, metaphase II oocytes; EGA, embryonic genome activation stage. Supplementary Table S3: the 200 most upregulated and downregulated in the BL compared with the MII stage in human. MII, metaphase II oocytes; BL, blastocysts. Supplementary Table S4: the 200 most upregulated and downregulated in the BL compared with the EGA stage in human. EGA, embryonic genome activation stage; BL, blastocysts. Supplementary Table S5: the 200 most downregulated in the EGA compared with the MII stage in mouse. MII, metaphase II oocytes; EGA, embryonic genome activation stage. Supplementary Table S6: the 200 most upregulated and downregulated in the BL compared with the MII stage in mouse. MII, metaphase II oocytes; BL, blastocyte. Supplementary Table S7: the 200 most upregulated and the 3 downregulated in the BL compared with the EGA stage in mouse. EGA, embryonic genome activation stage; BL, blastocyte. [file 5895628.f1.pdf]

Supplementary Table S1: Sequence of the forward and reverse primers used for the RT-qPCR analysis.

	Gene	Primers
Human	**BCL2L10**	GGATGGCTTTTGTCACTTCTTCAGG
		ATAATCGTGTCCAGAGATAAATGAAGGC
	**TNFRSF21**	AGAAGATTCGTGGGCTGATG
		CCCTTGTTCTTGTCCTGTGG
	**ENDOG**	CGACACGTTCTACCTGAGCA
		CTTGCCGATGACCTGGTACT
	**FGFR3**	AACGTGATGAAGATCGCAGA
		AGCGTGAAGATCTCCCAGAG
	**HPRT1**	GCCAGACTTTGTTGGATTTGA
		AAGCAGATGGCCACAGAACT
Mouse	**TGFB2**	TCGATAGCAAGGTTGTGAAA
		CGAGCTCTTCGCTTTTATTC
	**CASP3**	GATGGCTTGCCAGAAGATAC
		GCGAGTGAGAATGTGCATAA
	**TNFSF13B**	CTTTGCTATGGGTCATGTCA
		CGTGAAATCTGTGCATTCTC
	**TGFBR1**	GCCCCTGAAGTTCTAGATGA
		CATTTCTTCAACCGATGGAT
	**LBR**	CTTGGCAATGATCTTGGTTA
		GGAGGCTGTAGGTAAATGGA

Supplementary Table S2: The 200 most up- and down-regulated in the EGA compared with the MII stage in human.
MII, metaphase II oocytes; EGA, embryonic genome activation stage.

Gene ID	Gene Name	Fold Change	q-value(%)	Gene ID	Gene Name	Fold Change	q-value(%)
220184_at	NANOG	2577,9	0	202157_s_at	CUGBP2	-129,3	0
220513_at	C6orf148	1596,5	0	218006_s_at	ZNF22	-88,2	0,1150853
236914_at	AW080028	1424,6	0,1150853	224964_s_at	GNG2	-66,1	0
1553619_a_at	TRIM43	1373,4	0,043577	212966_at	HIC2	-64,2	0
1552456_a_at	MBD3L2	853,5	0,0332986	1556009_at	PEX13	-55,3	0
1554036_at	ZBTB24	695,4	0,8751441	203791_at	DMXL1	-54,9	0,1512685
228007_at	C6orf204	624,7	0,3380039	229606_at	Hs,634850	-51,1	0
214603_at	MAGEA2	567,1	0	222875_at	DHX33	-47,2	0
222867_s_at	MED31	482,6	0	230051_at	C10orf47	-46,1	0
216034_at	SUHW1	452,4	0	218170_at	ISOC1	-45,4	0
1552852_a_at	ZSCAN4	446,9	0	1554246_at	C1orf210	-44,6	0,043577
244206_at	ANUBL1	324,9	0	226572_at	SOCS7	-42,9	0
205899_at	CCNA1	317,1	0	239671_at	Hs,614930	-41,0	0,0509764
240301_at	DPPA2	297,8	0	209484_s_at	C1orf48	-40,8	0
201548_s_at	JARID1B	290,2	0	222673_x_at	TMEM57	-40,5	0
202499_s_at	SLC2A3	285,8	0,1512685	222895_s_at	BCL11B	-40,1	0
239155_at	LOC653108	283,7	0,0186691	229886_at	FLJ32363	-38,7	0
203957_at	E2F6	251,8	0,6288738	226038_at	LONRF1	-37,5	0,0332986
205966_at	TAF13	251,5	1,2299641	242702_at	H10659	-37,2	0,0509764
214097_at	RPS21	245,3	0,3380039	223249_at	CLDN12	-37,0	0
209942_x_at	MAGEA3	225,0	0,043577	219683_at	FZD3	-37,0	0
240318_at	AFMID	223,2	0,066989	1562762_at	C3orf56	-35,9	0,0332986
220941_s_at	C21orf91	213,9	0,043577	238444_at	ZNF618	-35,2	0,3380039
204124_at	AF146796	202,0	0,1150853	226910_at	Hs,432729	-35,1	0,1150853
213718_at	RBM4	199,9	0,2270814	226562_at	ZNF690	-34,8	0
222669_s_at	SBDS	195,9	1,2299641	229908_s_at	Hs,598368	-33,8	0,0212465
221791_s_at	CCDC72	188,9	0	239178_at	Hs,197018	-33,7	0
201483_s_at	SUPT4H1	188,4	0,1150853	234994_at	KIAA1913	-33,6	0
1559108_at	VPS53	184,0	1,2299641	202797_at	SACM1L	-32,2	0
207934_at	RFPL1	179,8	0,4862106	225112_at	ABI2	-31,7	0,043577
212022_s_at	MKI67	177,3	0	230288_at	AW418619	-31,5	0,0212465
201631_s_at	IER3	176,6	0,0332986	224048_at	USP44	-31,4	0
223000_s_at	F11R	176,5	0	215952_s_at	OAZ1	-30,9	0
230847_at	T79870	174,2	0,8751441	218622_at	NUP37	-30,6	0,0332986
233737_s_at	LOC284561	171,5	0	213626_at	CBR4	-30,6	0
1553697_at	C1orf96	169,2	0,0186691	208296_x_at	TNFAIP8	-30,2	0,0509764
206207_at	CLC	166,9	0,0899063	229085_at	LRRC3B	-29,7	0,8751441
213326_at	VAMP1	165,0	0,0332986	228293_at	DEPDC7	-29,5	0,0212465
222801_s_at	FLJ13195	161,7	0,0186691	1553064_at	H1FOO	-29,2	0,3380039
230362_at	INPP5F	158,0	0	235520_at	SUHW3	-28,7	0
200853_at	H2AFZ	149,5	0	224643_at	LOC133619	-28,5	0
230748_at	SLC16A6	148,4	0,1150853	242617_at	GSTZ1	-28,4	0
215227_x_at	ACP1	145,1	0	1554480_a_at	SVH	-28,3	0,066989
239127_at	Hs,446041	144,5	0,1512685	200958_s_at	SDCBP	-28,1	0
200085_s_at	TCEB2	143,3	0,8751441	235727_at	BTBD5	-28,1	0,0212465
216001_at	LOC390999	139,8	0,1512685	202615_at	GNAQ	-27,9	0
227724_at	LOC642351	139,4	0	226181_at	TUBE1	-27,8	0
63009_at	SHQ1	137,4	0,0899063	218361_at	GOLPH3L	-27,5	0,0212465
238912_x_at	C9orf85	135,5	0,0332986	227146_at	QSCN6L1	-27,3	0,066989
204379_s_at	FGFR3	133,6	0,2270814	225710_at	Hs,173030	-27,1	0,0509764
207227_x_at	RFPL2	133,6	0,043577	222603_at	KIAA1815	-27,1	0,0509764
203224_at	RFK	131,0	0,066989	212420_at	ELF1	-26,9	0,0509764
231339_at	TSPYL6	128,6	0,1512685	205964_at	ZNF426	-26,9	0
213550_s_at	AA993683	127,7	0,066989	212976_at	R41498	-26,8	0
207302_at	SGCG	123,8	0,2270814	208424_s_at	CIAPIN1	-26,7	0,0509764
221263_s_at	SF3B5	122,7	0,4862106	232204_at	EBF	-26,7	0
214882_s_at	SFRS2	119,7	0	217985_s_at	BAZ1A	-26,5	0
228977_at	IL17D	118,2	0,6288738	222848_at	FKSG14	-26,3	0
202431_s_at	MYC	117,4	0	209760_at	KIAA0922	-26,3	0,0212465
231568_at	RP6-166C19,1	114,2	0	204649_at	TROAP	-26,3	0,043577
238084_at	PCGF3	113,7	0	227801_at	TRIM59	-25,9	0,0899063
229864_at	TMEM103	112,3	0,1512685	208919_s_at	NADK	-25,6	0,1150853
214612_x_at	MAGEA6	111,7	0,0186691	225835_at	SLC12A2	-25,5	0,0212465

203917_at	CXADR	111,0	0,0332986	229174_at	AI688663	-25,3	0
222857_s_at	KCNMB4	109,7	0	236219_at	AI452512	-25,1	0,2270814
222666_s_at	RCL1	108,9	1,2299641	227454_at	TAOK1	-25,1	0,4862106
220399_at	FLJ22639	108,8	1,2299641	219274_at	TSPAN12	-24,9	0
242896_at	BF223302	107,7	0	234725_s_at	SEMA4B	-24,8	0,1150853
202391_at	BASP1	107,5	0	226273_at	LOC158563	-24,6	0
209430_at	BTAF1	104,4	0,043577	202412_s_at	USP1	-24,2	0,0332986
214960_at	API5	100,7	0,043577	202594_at	LEPROTL1	-24,1	0
204883_s_at	HUS1	100,0	0	205345_at	BARD1	-24,0	0
200734_s_at	ARF3	99,8	0	225961_at	KLHDC5	-23,9	0,0509764
243161_x_at	ZFP42	97,9	0,0509764	226800_at	KIAA1799	-23,7	0
225152_at	ZNF622	97,9	0	213729_at	PRPF40A	-23,5	0,0899063
207574_s_at	GADD45B	96,9	0,043577	235037_at	TMEM41A	-23,4	0
238077_at	KCTD6	96,8	0	230875_s_at	ATP11A	-23,3	0
1557257_at	BCL10	96,8	0,1512685	204759_at	RCBTB2	-23,0	0,0212465
213124_at	ZNF473	96,4	0,0899063	224681_at	GNA12	-23,0	0,1150853
230304_at	Hs,143408	95,6	0,1150853	214464_at	CDC42BPA	-22,9	0,0899063
217365_at	PRAMEF5	90,4	1,2299641	226050_at	TMCO3	-22,7	0
207438_s_at	RNUT1	90,3	0,0186691	1569302_at	KIAA1731	-22,7	0,1512685
239377_at	MGC11102	89,9	0,3380039	225761_at	PAPD4	-22,6	0
221970_s_at	NOL11	89,2	0	213249_at	FBXL7	-22,6	0,0332986
214183_s_at	TKTL1	86,9	2,4366888	222708_s_at	STX17	-22,2	0,066989
223096_at	NOP5/NOP58	85,4	0	41512_at	Hs,530940	-22,1	0,0212465
204233_s_at	CHKA	84,1	0,6288738	231920_s_at	CSNK1G1	-22,1	0,066989
209836_x_at	BOLA2	84,1	0,2270814	204057_at	IRF8	-22,1	0,4862106
201174_s_at	TERF2IP	83,5	0,2270814	1554176_a_at	C3orf33	-21,9	0
243475_at	CBL	81,7	0,066989	225361_x_at	RP11-308B5,5	-21,9	0
216302_at	HNRPC	81,0	0,4862106	212053_at	KIAA0251	-21,9	0,0899063
228875_at	C6orf189	79,8	0,6288738	228980_at	RFFL	-21,9	0,066989
31874_at	GAS2L1	79,6	0,1512685	234987_at	C20orf118	-21,9	0
222703_s_at	YRDC	78,6	0,1512685	218366_x_at	METT11D1	-21,8	0,1512685
223857_x_at	TMEM85	77,6	0	204554_at	PPP1R3D	-21,8	0,0212465
218379_at	RBM7	77,2	0,1512685	209649_at	STAM2	-21,8	0
226014_at	EIF3S5	77,0	0,0899063	230718_at	HSF5	-21,8	0,1512685
223773_s_at	C1orf79	75,9	0,1150853	201661_s_at	ACSL3	-21,8	0
235245_at	TMEM92	75,3	0	205251_at	PER2	-21,7	0,0212465
203094_at	MAD2L1BP	75,0	0	218474_s_at	KCTD5	-21,6	0
206626_x_at	SSX1	74,3	0,1512685	224650_at	MAL2	-21,6	0,0509764
217958_at	TRAPPC4	74,1	0	212074_at	UNC84A	-21,5	0
223391_at	SGPP1	74,1	0	221905_at	CYLD	-21,4	0,043577
210467_x_at	MAGEA12	74,0	0	1552664_at	FLCN	-21,2	0,0332986
213705_at	Hs,592466	73,7	0	208777_s_at	PSMD11	-21,1	0
224610_at	STX5	73,6	0	229114_at	Hs,632864	-20,9	0,0212465
205780_at	BIK	73,1	0,6288738	206536_s_at	BIRC4	-20,9	0,0332986
226565_at	TMEM99	73,0	0,066989	1555886_at	PDSS2	-20,7	0
205178_s_at	RBBP6	71,1	0,1150853	225864_at	FAM84B	-20,6	0,1150853
207828_s_at	CENPF	70,9	0,8751441	226026_at	DIRC2	-20,6	0,1150853
209757_s_at	MYCN	70,8	0,0509764	206138_s_at	PIK4CB	-20,3	0,1150853
1569077_x_at	FLJ16287	69,9	1,2299641	201411_s_at	PLEKHB2	-20,2	0
226419_s_at	FLJ44342	69,7	0	225941_at	EIF4E3	-20,1	0
218672_at	SCNM1	69,6	0,0186691	206588_at	DAZL	-19,9	0
209593_s_at	TOR1B	69,5	0	221064_s_at	C16orf28	-19,8	0,0212465
209007_s_at	C1orf63	69,0	0,0186691	226154_at	DNM1L	-19,8	0,043577
214395_x_at	EEF1D	68,9	0	226383_at	C11orf46	-19,2	0,1512685
205031_at	EFNB3	68,0	0,8751441	224937_at	PTGFRN	-19,1	0,0212465
221501_x_at	LOC339047	67,9	0	235931_at	AI332764	-18,7	0
1555301_a_at	DIP2A	67,0	0,4862106	228390_at	Hs,594773	-18,3	0
203544_s_at	STAM	65,8	0	229355_at	Hs,595430	-18,3	0
209482_at	POP7	63,7	0	221675_s_at	CHPT1	-18,2	0,4862106
218940_at	C14orf138	63,6	0,043577	201841_s_at	HSPB1	-18,1	0,6288738
229790_at	TERF2	63,6	0,043577	226284_at	ZBTB2	-18,0	0
229106_at	DYNLL2	63,0	0,0186691	213106_at	AI769688	-17,8	0
205895_s_at	NOLC1	62,9	0	212676_at	Hs,113577	-17,7	0,1150853
205747_at	CBLN1	62,5	0,1512685	201301_s_at	ANXA4	-17,6	0,0212465
228690_s_at	NDUFA11	62,2	0,1512685	220145_at	ASAP	-17,6	0,0509764
223787_s_at	C3orf19	61,5	0,0332986	226666_at	GPR135	-17,3	0,0212465
1555967_at	Hs,21423	61,1	1,2299641	231810_at	BRI3BP	-17,2	0,0332986

224444_s_at	C1orf97	60,8	0,2270814	210993_s_at	SMAD1	-17,1	0
218882_s_at	WDR3	60,5	0	212604_at	MRPS31	-17,1	0
200886_s_at	PGAM1	60,1	0,0332986	218218_at	DIP13B	-17,0	0
214149_s_at	ATP6V0E	59,5	0	218859_s_at	C20orf6	-16,9	0,0212465
226300_at	MED19	59,2	0,1512685	227139_s_at	HPS3	-16,9	0,4862106
1554245_x_at	ARL17P1	58,7	0	213064_at	ZC3H14	-16,9	0
214224_s_at	PIN4	58,2	0	223342_at	RRM2B	-16,8	0
57703_at	SENP5	58,0	0	204403_x_at	KIAA0738	-16,7	0,066989
213826_s_at	AA292281	57,8	0	219637_at	ARMC9	-16,6	0,0899063
214661_s_at	C4orf9	57,6	0	219972_s_at	C14orf135	-16,6	0,1512685
208735_s_at	CTDSP2	56,3	0,0186691	213372_at	PAQR3	-16,5	0,066989
204030_s_at	SCHIP1	55,6	0	223693_s_at	FLJ10324	-16,5	0,2270814
221536_s_at	LSG1	55,6	0,0899063	203357_s_at	CAPN7	-16,5	0,043577
203847_s_at	AKAP8	55,6	0	216060_s_at	DAAM1	-16,4	0
1553103_at	NFX1	54,7	0,4862106	205883_at	ZBTB16	-16,4	0,3380039
228135_at	C1orf52	53,5	0	226541_at	FBXO30	-16,3	0
200664_s_at	DNAJB1	52,7	0	228401_at	ATAD2	-16,3	0
241937_s_at	WDR4	51,8	0,0899063	210948_s_at	LEF1	-16,2	0,1512685
218188_s_at	TIMM13	51,5	0,4862106	235241_at	FLJ90709	-16,2	0
202233_s_at	UQCRH	51,3	0,043577	213899_at	METAP2	-16,2	0,0509764
203705_s_at	FZD7	51,3	0,1150853	218698_at	APIP	-16,0	0,1150853
225200_at	ZCSL2	51,2	2,4366888	226751_at	C2orf32	-16,0	0,1512685
223560_s_at	PRO1853	50,9	0,066989	1562386_s_at	ZNF501	-16,0	0,0509764
210431_at	ALPPL2	50,8	0,0186691	220321_s_at	FLJ13646	-16,0	0,3380039
231118_at	ANKRD35	50,8	0,043577	201502_s_at	NFKBIA	-16,0	0,0332986
218238_at	GTPBP4	50,6	0,0509764	219736_at	TRIM36	-15,9	0
209418_s_at	THOC5	50,5	0,1150853	231862_at	Hs,349283	-15,9	0
238066_at	RBP7	50,5	0,3380039	233819_s_at	ZNF294	-15,9	0,3380039
231119_at	RFC3	50,3	0	202368_s_at	TRAM2	-15,9	0,1150853
226720_at	MST101	50,2	0,0186691	201752_s_at	ADD3	-15,8	0,0509764
46665_at	SEMA4C	49,7	0,1150853	212812_at	SERINC5	-15,7	0,0509764
219138_at	RPL14	49,4	0	225847_at	AADACL1	-15,7	0,1512685
244189_at	KIAA1648	49,3	0,0186691	202666_s_at	ACTL6A	-15,5	0
204285_s_at	PMAIP1	49,1	1,7491695	213216_at	OTUD3	-15,3	0,1512685
204833_at	ATG12	49,1	0	212083_at	TEX261	-15,3	0
215093_at	NSDHL	49,0	0	201552_at	LAMP1	-15,2	0,0332986
205217_at	TIMM8A	49,0	0,0899063	223065_s_at	STARD3NL	-15,1	0,066989
217883_at	C2orf25	48,9	0	234986_at	Hs,596052	-15,0	0,0509764
1558620_at	ZNF621	48,4	0,1150853	201880_at	ARIH1	-15,0	0,1150853
235296_at	EIF5A2	48,2	2,4366888	203306_s_at	SLC35A1	-14,8	0
232097_at	C14orf92	48,2	0,1512685	47608_at	TJAP1	-14,8	0,0212465
222380_s_at	PDCD6	48,1	0,0899063	226283_at	WDR51B	-14,7	0
227909_at	MGC39606	48,0	1,2299641	1565834_a_at	BU177699	-14,6	0,043577
213847_at	PRPH	47,7	0,1512685	215380_s_at	C7orf24	-14,6	0
213554_s_at	CDV3	47,6	0	202289_s_at	TACC2	-14,6	0,043577
1562722_at	FLJ40296	47,3	0,0332986	226592_at	LOC286334	-14,6	0,043577
228301_x_at	NDUFB10	47,1	0,0186691	224436_s_at	NIPSNAP3A	-14,5	0
208312_s_at	PRAMEF1	47,1	0,6288738	240770_at	PRP2	-14,5	0,4862106
236221_at	AP4B1	46,9	0,3380039	222544_s_at	WHSC1L1	-14,4	0,3380039
201323_at	EBNA1BP2	46,4	0,066989	224695_at	C2orf29	-14,2	0
244139_s_at	AI435073	46,2	0,1150853	218297_at	C10orf97	-14,1	0,1150853
208720_s_at	RNPC2	45,9	0,0186691	204674_at	LRMP	-14,1	0,3380039
225687_at	FAM83D	45,9	0	209055_s_at	CDC5L	-14,1	0,066989
207936_x_at	RFPL3	45,8	0,2270814	223309_x_at	IPLA2(GAMMA)	-14,0	0,0509764
226175_at	TTC9C	45,8	0,0186691	233841_s_at	SUDS3	-14,0	0
218189_s_at	NANS	45,8	0	208723_at	USP11	-14,0	0,043577
210757_x_at	DAB2	45,3	0,8751441	235103_at	MAN2A1	-14,0	0,1150853
219681_s_at	RAB11FIP1	44,7	0,0899063	221904_at	C3orf40	-14,0	0,0899063
205545_x_at	DNAJC8	44,5	0	243495_s_at	Hs,624172	-14,0	0
222040_at	HNRPA1	44,5	0,043577	202016_at	MEST	-14,0	0,066989
219904_at	ZSCAN5	44,4	0,0186691	219588_s_at	LUZP5	-13,8	0,1512685
223428_s_at	KIAA1160	44,4	0,1150853	204706_at	INPP5E	-13,8	0,1150853
227278_at	AI056692	44,2	0	221575_at	SCLY	-13,8	0,1150853
201573_s_at	ETF1	43,5	0,1150853	205190_at	PLS1	-13,7	0,0212465
227603_at	Hs,640243	43,3	0,6288738	232530_at	LOC652226	-13,7	0,4862106
210790_s_at	SAR1A	43,2	0,2270814	228201_at	ARL13B	-13,7	0
225831_at	LUZP1	42,7	0,066989	225185_at	MRAS	-13,7	0,0212465

226297_at	Hs,594939	42,6	0,0509764
202464_s_at	PFKFB3	42,1	0
217980_s_at	MRPL16	41,8	0

215933_s_at	HHEX	-13,7	0
203825_at	BRD3	-13,7	0,0509764
223358_s_at	Hs,527119	-13,6	0

Supplementary Table S3: The 200 most up- and down-regulated in the BL compared with the MII stage in human.
MII, metaphase II oocytes; BL, blastocysts.

Gene ID	Gene Name	Fold Change	q-value(%)
201596_x_at	KRT18	2240,8	0
227048_at	LAMA1	1670,6	0
209262_s_at	NR2F6	1456,1	0
220139_at	DNMT3L	1258,9	0
220513_at	C6orf148	899,2	0,09957446
224972_at	C20orf52	862,2	0
204379_s_at	FGFR3	817,8	0,01715523
201650_at	KRT19	780,2	0,00679705
200085_s_at	TCEB2	753,8	0
205081_at	CRIP1	711,4	0,03875166
202023_at	EFNA1	682,3	0,05893542
205450_at	PHKA1	676,9	0,00382982
223689_at	IGF2BP1	612,1	0
202286_s_at	TACSTD2	580,7	0,01715523
202499_s_at	SLC2A3	575,6	0
202800_at	SLC1A3	541,0	0
209604_s_at	GATA3	526,5	0
204351_at	S100P	488,2	0,05893542
225660_at	SEMA6A	396,9	0,00382982
205967_at	HIST1H4C	378,9	0
221263_s_at	SF3B5	364,6	0
215729_s_at	VGLL1	349,3	0,03875166
229125_at	ANKRD38	340,1	0,05893542
216379_x_at	CD24	323,5	0,00679705
213550_s_at	AA993683	311,5	0
45288_at	ABHD6	297,6	0,03875166
210431_at	ALPPL2	296,1	0,15345984
224232_s_at	PX19	288,7	0
202598_at	S100A13	278,5	0,00382982
211162_x_at	SCD	275,1	0,01361431
229332_at	GLOXD1	269,8	0,00382982
233396_s_at	CSRP2BP	266,1	0
232191_at	C21orf105	250,1	0
228690_s_at	NDUFA11	248,1	0
224932_at	C22orf16	241,6	0,02565228
201278_at	DAB2	230,1	0,00679705
212761_at	TCF7L2	227,0	0,00912068
209735_at	ABCG2	217,1	0
222435_s_at	UBE2J1	212,3	0
202626_s_at	LYN	211,7	0,02565228
200844_s_at	PRDX6	206,2	0
223000_s_at	F11R	204,7	0
209710_at	GATA2	204,0	0,01361431
203709_at	PHKG2	201,6	0,38816402
39248_at	AQP3	192,3	0,00912068
33322_i_at	SFN	191,1	0,02565228
203219_s_at	APRT	190,7	0,00382982
46665_at	SEMA4C	190,0	0,00382982
204883_s_at	HUS1	188,8	0,00382982
221501_x_at	LOC339047	187,5	0
212444_at	Hs.632997	185,7	0,03875166
216338_s_at	YIPF3	183,1	0
221791_s_at	CCDC72	182,1	0
210367_s_at	PTGES	181,8	0,00382982
214285_at	FABP3	180,9	0,09957446
219138_at	RPL14	178,3	0
226214_at	MIR16	174,6	0
214097_at	RPS21	169,8	0
209836_x_at	BOLA2	164,9	0,00382982
204538_x_at	NPIP	159,8	0,00382982
218454_at	FLJ22662	155,6	0
223368_s_at	C9orf32	155,0	0

Gene ID	Gene Name	Fold Change	q-value(%)
230288_at	AW418619	-589,1	0,00679705
220766_at	BTG4	-588,8	0
219932_at	SLC27A6	-504,9	0
234994_at	KIAA1913	-426,7	0
230645_at	FRMD3	-361,4	0
206588_at	DAZL	-349,0	0
226751_at	C2orf32	-337,8	0,03875166
227021_at	AOF1	-318,9	0
236491_at	BCL2L10	-298,6	0,01361431
211915_s_at	LOC649679	-268,9	0,05893542
217047_s_at	FAM13A1	-259,4	0
205190_at	PLS1	-248,4	0,00483604
206404_at	FGF9	-231,1	0,01715523
244475_at	LOC646951	-226,6	0,00912068
1562762_at	C3orf56	-205,7	0,00679705
203355_s_at	PSD3	-202,8	0
205529_s_at	RUNX1T1	-194,4	0
229738_at	DNAH10	-166,1	0,01361431
215933_s_at	HHEX	-152,3	0
232720_at	LRRN6C	-148,0	0
231386_at	FLJ30851	-145,5	0,01715523
223533_at	LRRC8C	-140,4	0,02565228
227022_at	GNPDA2	-138,9	0,00483604
224800_at	WDFY1	-132,2	0
215321_at	RPIB9	-131,3	0,01361431
226907_at	PPP1R14C	-117,9	0,01715523
216060_s_at	DAAM1	-117,3	0
212943_at	KIAA0528	-114,5	0,00483604
214053_at	Hs.587035	-114,4	0
222835_at	THSD4	-112,7	0,00912068
1553527_at	NALP9	-112,1	0
1554300_a_at	LOC136306	-112,1	0,00679705
211596_s_at	LRIG1	-111,0	0,02565228
229606_at	Hs.634850	-110,9	0,00483604
223475_at	CRISPLD1	-110,1	0
220135_s_at	SLC7A9	-106,6	0,02565228
204759_at	RCBTB2	-105,0	0,00483604
227794_at	GLYATL1	-103,9	0,00483604
232692_at	TDRD6	-96,0	0,00679705
222291_at	AI478795	-95,0	0,01361431
226117_at	TIFA	-93,5	0,00483604
243346_at	LMOD3	-92,1	0,02565228
201242_s_at	ATP1B1	-91,4	0
212074_at	UNC84A	-87,8	0
219806_s_at	C11orf75	-86,7	0,00483604
227997_at	IL17RD	-85,5	0
219034_at	PARP16	-85,3	0,01715523
231474_at	ECAT1	-84,7	0,15345984
218311_at	MAP4K3	-84,0	0
1555775_a_at	ZAR1	-84,0	0
213228_at	PDE8B	-80,6	0,00483604
205338_s_at	DCT	-80,3	0,01361431
205302_at	IGFBP1	-80,3	0
209392_at	ENPP2	-80,3	0
220653_at	ZIM2	-79,1	0
223374_s_at	B3GALNT1	-77,4	0
229085_at	LRRC3B	-76,4	0,38816402
226271_at	GDAP1	-74,4	0
213353_at	ABCA5	-71,6	0,00912068
225101_s_at	SNX14	-71,0	0,00679705
223503_at	DKFZP566N034	-70,7	0
209804_at	DCLRE1A	-70,3	0,00912068

213720_s_at	SMARCA4	152,4	0
222392_x_at	PERP	150,2	0
50221_at	TFEB	150,0	1,13755209
207357_s_at	GALNT10	144,6	0,01715523
201483_s_at	SUPT4H1	143,7	0
222496_s_at	FLJ20273	142,1	0,00382982
219155_at	PITPNC1	141,5	0,00912068
212048_s_at	YARS	140,9	0
225516_at	SLC7A2	139,9	0,00382982
203234_at	UPP1	139,9	0
209191_at	TUBB6	139,6	0
202830_s_at	SLC37A4	138,7	0
208613_s_at	FLNB	138,0	0,00679705
232164_s_at	EPPK1	137,1	0,01361431
218357_s_at	TIMM8B	137,0	0
31874_at	GAS2L1	135,9	0,02565228
201757_at	NDUFS5	129,3	0
243161_x_at	ZFP42	127,1	0,00382982
224646_x_at	H19	126,9	0,67162846
208510_s_at	PPARG	125,4	0,01715523
240301_at	DPPA2	125,2	0,00382982
201275_at	FDPS	124,5	0,00382982
228462_at	IRX2	122,8	0
224233_s_at	MSTO1	122,4	0
228301_x_at	NDUFB10	121,4	0
212424_at	PDCD11	121,3	0
203047_at	STK10	120,7	0,15345984
218672_at	SCNM1	120,5	0,00382982
223773_s_at	C1orf79	119,4	0,00382982
204416_x_at	APOC1	118,8	0,00912068
202306_at	POLR2G	117,8	0
212203_x_at	IFITM3	116,9	0,00382982
200824_at	GSTP1	116,7	0,01361431
200820_at	PSMD8	116,2	0
219681_s_at	RAB11FIP1	115,8	0
218856_at	TNFRSF21	115,0	0,09957446
212411_at	IMP4	111,8	0
221779_at	MICAL-L1	110,6	0,01361431
229498_at	Hs.291319	110,4	0,01715523
224864_at	SRA1	110,0	0
1559946_s_at	RUVBL2	108,8	0
217221_x_at	RBM10	108,0	0,01361431
209757_s_at	MYCN	107,5	0
229074_at	Hs.598990	105,4	0
202391_at	BASP1	105,3	0
218188_s_at	TIMM13	104,3	0,00382982
223075_s_at	C9orf58	103,5	0,05893542
225321_s_at	PILRB	103,3	0
226419_s_at	FLJ44342	101,7	0,00382982
202937_x_at	CTA-126B4.3	101,1	0,02565228
227829_at	GYLTL1B	100,2	0,00382982
202790_at	CLDN7	97,7	0,00382982
209482_at	POP7	97,6	0
210517_s_at	AKAP12	96,0	0,01361431
204044_at	QPRT	95,9	0,00912068
203957_at	E2F6	95,8	0
211025_x_at	COX5B	95,3	0
203114_at	SSSCA1	95,0	0
231195_at	FLJ44186	94,8	0,00382982
212022_s_at	MKI67	94,5	0
215227_x_at	ACP1	94,4	0
1555895_at	DNM2	93,3	0
218385_at	MRPS18A	92,8	0
203416_at	CD53	92,4	0,01715523
226553_at	TMPRSS2	91,3	0,15345984
201573_s_at	ETF1	91,2	0

227609_at	EPSTI1	-69,7	0
223162_s_at	LCHN	-69,5	0,00912068
235736_at	Hs.178144	-69,5	0,00483604
218793_s_at	SCML1	-69,4	0,00912068
221314_at	GDF9	-68,9	0
1565579_at	Hs.611748	-68,7	0,02565228
220116_at	KCNN2	-68,3	0,00912068
237254_at	SLC5A11	-67,3	0,09957446
229886_at	FLJ32363	-66,7	0
203531_at	CUL5	-66,6	0,02565228
226572_at	SOCS7	-66,2	0
228260_at	ELAVL2	-65,9	0,01715523
241360_at	CCDC15	-65,8	0,00912068
229355_at	Hs.595430	-64,7	0
1553086_at	C11orf40	-64,5	0
232087_at	CXorf23	-63,2	0
229908_s_at	Hs.598368	-61,5	0,00483604
228454_at	MLR2	-61,3	0
230889_at	LOC645321	-61,3	0,67162846
222552_at	GOLT1B	-61,3	0
223693_s_at	FLJ10324	-60,7	0,05893542
224452_s_at	MGC12966	-60,3	0,01361431
213106_at	AI769688	-59,7	0
204776_at	THBS4	-59,6	0,00483604
209884_s_at	SLC4A7	-59,1	0
226181_at	TUBE1	-58,7	0
210495_x_at	FN1	-58,5	0,03875166
213249_at	FBXL7	-58,5	0,00679705
219515_at	PRDM10	-58,4	0
239061_at	TPRXL	-58,3	0,02565228
204284_at	PPP1R3C	-57,8	1,9395689
226562_at	ZNF690	-57,5	0
230626_at	TSPAN12	-57,3	0,01715523
203011_at	IMPA1	-57,3	0
226273_at	LOC158563	-55,9	0
229091_s_at	CCNJ	-55,9	0
204137_at	GPR137B	-55,9	0,02565228
202388_at	RGS2	-55,4	0,01361431
202797_at	SACM1L	-55,0	0,00483604
229459_at	FAM19A5	-54,5	0,01361431
1552531_a_at	NALP11	-54,4	0,00483604
204057_at	IRF8	-54,3	0,26525376
206613_s_at	TAF1A	-54,2	0,02565228
230543_at	AI761675	-54,0	0,38816402
1556096_s_at	UNC13C	-53,5	0
225655_at	UHRF1	-53,0	0
218191_s_at	LMBRD1	-52,6	0,00483604
205964_at	ZNF426	-52,5	0
242334_at	NALP4	-52,4	0,01361431
204980_at	CLOCK	-52,0	0
221064_s_at	C16orf28	-51,7	0,00483604
238584_at	IQCA	-51,6	0,00912068
223342_at	RRM2B	-51,1	0
1555883_s_at	SPIN3	-50,9	0
203037_s_at	MTSS1	-50,7	0,15345984
219947_at	CLEC4A	-49,9	0,00912068
51158_at	LOC400451	-49,8	0,05893542
218297_at	C10orf97	-48,3	0,02565228
223249_at	CLDN12	-47,5	0,00483604
226980_at	DEPDC1B	-47,3	0
1561673_at	Hs.434703	-47,1	0
210314_x_at	TNFSF13	-47,0	0
1557085_at	TMEM122	-46,9	0,00679705
207443_at	NR2E1	-46,5	0,03875166
227475_at	FOXQ1	-46,2	0
203357_s_at	CAPN7	-45,8	0,00912068

227998_at	S100A16	91,2	0
226296_s_at	MRPS15	89,7	0
200005_at	EIF3S7	89,2	0
209219_at	RDBP	87,3	0
219648_at	DSU	86,9	0,01361431
219117_s_at	FKBP11	86,8	0,00679705
210087_s_at	MPZL1	86,7	0
218408_at	TIMM10	86,6	0
200029_at	RPL19	86,4	0
201998_at	ST6GAL1	86,2	0
208700_s_at	TKT	86,1	0
226213_at	ERBB3	85,4	1,13755209
200826_at	SNRPD2	84,8	0
231236_at	ZFP57	83,5	0
203663_s_at	COX5A	83,1	0
212270_x_at	RPL17	83,0	0
215230_x_at	EIF3S8	83,0	0
204141_at	TUBB2A	82,6	0,01715523
225150_s_at	RTKN	82,5	0,00382982
228303_at	Hs.505575	81,7	0,05893542
228654_at	RP11-93B10.1	80,5	0
202233_s_at	UQCRH	80,0	0
221245_s_at	C2orf31	79,6	0
211730_s_at	POLR2L	79,2	0
201903_at	UQCRC1	79,1	0
210381_s_at	CCKBR	78,9	0,01361431
202857_at	TMEM4	78,7	0
204237_at	GULP1	78,3	0
225739_at	RAB11FIP4	78,2	0,00679705
219735_s_at	TFCP2L1	77,4	0,02565228
214661_s_at	C4orf9	77,3	0
1557094_at	Hs.626656	77,3	0,00679705
219646_at	FLJ20186	77,0	0,00382982
200012_x_at	RPL21	76,9	0
225967_s_at	LOC284184	76,3	0
230972_at	ANKRD9	76,2	0,00912068
213460_x_at	NSUN5C	76,1	0,00382982
223391_at	SGPP1	75,7	0
225788_at	C6orf153	75,3	0
201244_s_at	RAF1	75,2	0
224469_s_at	C14orf151	75,1	0
209122_at	ADFP	74,8	0,03875166
218278_at	WDR74	74,5	0
201428_at	CLDN4	74,4	0,01361431
1558007_s_at	LCMT1	74,3	1,9395689
211986_at	AHNAK	74,1	0,00382982
58696_at	EXOSC4	74,0	0
229711_s_at	MGC5370	73,2	0,01715523
223207_x_at	PHPT1	72,7	0,01715523
201066_at	CYC1	72,7	0
213348_at	CDKN1C	71,9	0,67162846
205829_at	HSD17B1	71,1	0
218571_s_at	CHMP4A	70,8	0
209418_s_at	THOC5	70,7	0
218281_at	MRPL48	70,5	0
203025_at	ARD1A	70,4	0
223839_s_at	Hs.597496	70,3	0
203782_s_at	POLRMT	70,1	0
217755_at	HN1	69,4	0
224301_x_at	H2AFJ	68,7	0,01715523
201416_at	SOX4	68,7	0,00382982
207585_s_at	RPL36AL	68,6	0
212116_at	RFP	68,2	0
224610_at	STX5	67,9	0
31845_at	ELF4	67,9	0,01361431
218526_s_at	RANGNRF	67,8	0

238944_at	Hs.623967	-45,6	0,09957446
218543_s_at	PARP12	-45,5	0,03875166
206794_at	ERBB4	-45,0	0,15345984
214769_at	Hs.592809	-44,5	0
236347_at	MMAA	-44,0	0,00483604
226925_at	ACPL2	-43,9	0
229475_at	MAEL	-43,8	0
203156_at	AKAP11	-43,7	0,01361431
202918_s_at	PREI3	-43,6	0
219683_at	FZD3	-43,5	0
233903_s_at	SGEF	-43,5	0,00679705
229105_at	GPR39	-43,3	0,00679705
226956_at	LOC400924	-43,3	0
204900_x_at	SAP30	-43,3	0,01361431
1555396_s_at	LOC340602	-43,2	0,01715523
207819_s_at	ABCB4	-42,8	1,13755209
228927_at	ZNF397	-42,8	0
204221_x_at	GLIPR1	-42,8	0
232422_at	RP11-151A6.2	-42,7	0,00912068
222437_s_at	VPS24	-42,5	0,00483604
227539_at	Hs.596900	-42,1	0,00912068
231731_at	OTX2	-42,1	0
222550_at	ARMC1	-42,0	0
227454_at	TAOK1	-41,5	0,15345984
204554_at	PPP1R3D	-41,3	0,00483604
1552405_at	NALP5	-41,3	0,00483604
227692_at	GNAI1	-41,3	0
213238_at	ATP10D	-41,2	0,00679705
218871_x_at	GALNACT-2	-41,1	0
236717_at	LOC165186	-41,0	0,00912068
218247_s_at	RKHD2	-40,7	0
238890_at	AI791303	-40,6	0
230300_at	Hs.586550	-40,5	0,01361431
236219_at	AI452512	-40,5	0,05893542
213996_at	YPEL1	-40,5	0
202323_s_at	ACBD3	-40,5	0,05893542
213922_at	TTBK2	-40,5	0,01361431
214451_at	TFAP2B	-40,1	0
219362_at	MAK10	-39,8	0,01361431
222361_at	LOC643224	-39,6	0,02565228
209566_at	INSIG2	-39,6	0
209043_at	PAPSS1	-38,8	0
233841_s_at	SUDS3	-38,6	0
201865_x_at	NR3C1	-38,3	0,00679705
225706_at	GLCCI1	-37,8	0,01361431
231944_at	ERO1LB	-37,8	0
225387_at	TSPAN5	-37,6	0
214683_s_at	CLK1	-37,6	0
228980_at	RFFL	-37,1	0,01715523
201841_s_at	HSPB1	-36,7	0,26525376
1562209_at	LOC285429	-36,7	0
213216_at	OTUD3	-36,4	0,03875166
209631_s_at	GPR37	-36,3	0
239642_at	Hs.593807	-36,3	0,00679705
1555074_a_at	KCNH5	-36,2	0,03875166
201847_at	LIPA	-36,1	0,00679705
225710_at	Hs.173030	-35,8	0,01715523
201133_s_at	PJA2	-35,7	0,01361431
244317_at	KIAA1324L	-35,7	0
209706_at	NKX3-1	-35,7	0,15345984
200914_x_at	KTN1	-35,7	0
213331_s_at	NEK1	-35,6	0,00483604
231448_at	Tenr	-35,6	0
218170_at	ISOC1	-35,5	0
227806_at	MGC17624	-35,5	0,05893542
203810_at	DNAJB4	-35,4	0

200853_at	H2AFZ	67,4	0,00382982
225152_at	ZNF622	67,0	0
38157_at	DOM3Z	67,0	0,03875166
227118_s_at	MRPS26	66,4	0,00679705
226014_at	EIF3S5	66,4	0
204309_at	CYP11A1	66,1	0,05893542

235588_at	ESCO2	-35,4	0,02565228
202850_at	ABCD3	-35,3	0
235828_at	MGC21644	-35,2	0,01361431
204369_at	PIK3CA	-35,2	0,09957446
227204_at	PARD6G	-35,2	0,00483604
1559954_s_at	DDX42	-34,9	0,01361431

Supplementary Table S4: The 200 most up- and down-regulated in the BL compared with the EGA stage in human.
EGA, embryonic genome activation stage; BL, blastocysts.

Gene ID	Gene Name	Fold Change	q-value(%)
201596_x_at	KRT18	583,4	0
216379_x_at	CD24	130,0	0,01763649
220139_at	DNMT3L	117,2	0
202286_s_at	TACSTD2	102,4	0,05147485
200832_s_at	SCD	101,8	0
209710_at	GATA2	96,5	0,02844238
225520_at	MTHFD1L	86,1	0,01763649
212481_s_at	TPM4	82,9	0,02844238
223168_at	RHOU	80,6	0
201903_at	UQCRC1	80,5	0
223062_s_at	PSAT1	75,9	0,18873999
201066_at	CYC1	75,8	0
228975_at	SP6	72,5	1,61463267
200872_at	S100A10	70,1	0
205967_at	HIST1H4C	69,5	0
201275_at	FDPS	68,9	0
200824_at	GSTP1	65,4	0,02844238
202090_s_at	UQCR	65,3	0,01763649
209735_at	ABCG2	63,9	0
205450_at	PHKA1	63,8	0
203665_at	HMOX1	62,2	0,02844238
202626_s_at	LYN	58,5	0,10846029
223207_x_at	PHPT1	57,1	0,05235772
218721_s_at	C1orf27	53,9	0,0753266
209604_s_at	GATA3	53,3	0
203108_at	GPRC5A	52,4	0,34371104
202598_at	S100A13	52,3	0,01763649
222986_s_at	SCOTIN	50,7	0
201278_at	DAB2	48,7	0,01763649
209146_at	SC4MOL	45,9	0,02844238
230863_at	Hs,595398	45,4	0
201063_at	RCN1	45,3	0
227998_at	S100A16	43,0	0
210367_s_at	PTGES	42,1	0
225516_at	SLC7A2	40,7	0,01763649
222435_s_at	UBE2J1	40,6	0
220525_s_at	AUP1	40,4	0
201757_at	NDUFS5	39,8	0
227829_at	GYLTL1B	39,4	0
209265_s_at	METTL3	39,1	0
201998_at	ST6GAL1	39,0	0
210087_s_at	MPZL1	38,0	0
212256_at	GALNT10	37,5	0,02844238
212137_at	LARP1	37,3	0
201135_at	ECHS1	37,0	0
209682_at	CBLB	36,8	0,6041608
203098_at	CDYL	36,4	0
208700_s_at	TKT	36,3	0
227042_at	LOC150223	36,1	0
221646_s_at	ZDHHC11	36,1	0,01763649
224847_at	CDK6	35,9	0,05147485
222392_x_at	PERP	35,8	0
212048_s_at	YARS	33,9	0
208710_s_at	AP3D1	33,4	0
203416_at	CD53	32,9	0,05147485
201250_s_at	SLC2A1	32,6	0,01763649
209120_at	NR2F2	32,0	1,14220607
201013_s_at	PAICS	31,8	0
212372_at	MYH10	31,6	0
232165_at	AL137725	31,2	0
217874_at	SUCLG1	31,0	0
200806_s_at	HSPD1	30,3	0,10846029

Gene ID	Gene Name	Fold Change	q-value(%)
1553619_a_at	TRIM43	-563,5	0,03145261
205899_at	CCNA1	-214,8	0
206140_at	LHX2	-212,5	0,03145261
244206_at	ANUBL1	-176,2	0
1552405_at	NALP5	-118,2	0,03145261
225081_s_at	CDCA7L	-116,8	0,46529687
1552456_a_at	MBD3L2	-113,7	0,02177985
209160_at	AKR1C3	-110,7	0
1559108_at	VPS53	-108,3	0,6041608
216034_at	SUHW1	-107,3	0
230748_at	SLC16A6	-105,4	0,0753266
1570337_at	FIGLA	-101,9	0,02177985
232692_at	TDRD6	-101,5	0,05147485
207443_at	NR2E1	-98,7	0,23707459
230626_at	TSPAN12	-97,3	1,14220607
231756_at	ZP4	-96,4	0
236914_at	AW080028	-81,4	0,10846029
214603_at	MAGEA2	-77,9	0
1552531_a_at	NALP11	-76,0	0
237131_at	LOC645469	-75,5	0,13454973
207936_x_at	RFPL3	-73,4	0,13454973
229105_at	GPR39	-72,3	0,13454973
226117_at	TIFA	-70,1	0,02177985
219229_at	SLCO3A1	-69,5	0,05147485
219932_at	SLC27A6	-69,2	0,46529687
217365_at	PRAMEF5	-68,2	0,81241439
216001_at	LOC390999	-62,1	0,13454973
214612_x_at	MAGEA6	-61,9	0,02177985
1556096_s_at	UNC13C	-60,7	0
234393_at	HDAC9	-60,3	0,05235772
204124_at	AF146796	-59,2	0,0753266
242334_at	NALP4	-59,1	0
231118_at	ANKRD35	-56,2	0,03145261
205747_at	CBLN1	-55,7	0,10846029
209942_x_at	MAGEA3	-55,6	0,03145261
239127_at	Hs,446041	-55,0	0,13454973
206404_at	FGF9	-54,9	1,14220607
226271_at	GDAP1	-54,6	0
239061_at	TPRXL	-54,5	0
1557544_at	C10orf80	-54,0	0
236117_at	Hs,42747	-51,9	0
236800_at	C10orf49	-51,1	0,23707459
1558111_at	MBNL1	-51,0	0,6041608
216302_at	HNRPC	-50,3	0,23707459
220766_at	BTG4	-49,0	0,05147485
230968_at	Hs,444785	-48,0	0,13454973
229738_at	DNAH10	-47,9	0,6041608
220410_s_at	CAMSAP1	-47,6	0,10846029
44783_s_at	HEY1	-45,7	0,10846029
205113_at	NEF3	-45,6	0,0753266
237464_at	IMAA	-45,6	0,23707459
204920_at	CPS1	-44,5	0
231448_at	Tenr	-44,1	0
1554671_a_at	SRRM2	-43,1	0,46529687
238021_s_at	LOC643911	-42,5	0
1557146_a_at	FLJ32252	-42,0	0,18873999
202388_at	RGS2	-41,5	0
227794_at	GLYATL1	-41,3	0
220135_s_at	SLC7A9	-40,2	0
209994_s_at	ABCB1	-40,1	0,13454973
222835_at	THSD4	-39,2	0,02177985
204784_s_at	MLF1	-38,9	0

204044_at	QPRT	30,0	0,02844238	230450_at	AI806866	-38,7	0,34371104
225739_at	RAB11FIP4	29,2	0,01763649	226254_s_at	KIAA1430	-38,5	0,10846029
218639_s_at	ZXDC	28,4	0,34371104	239155_at	LOC653108	-37,9	0,02177985
213969_x_at	RPL29	28,0	0	219352_at	HERC6	-37,8	0,03145261
226538_at	MAN2A1	27,3	0	240318_at	AFMID	-37,0	0,05147485
212115_at	C16orf34	26,8	0	205011_at	LOH11CR2A	-36,8	0,23707459
224972_at	C20orf52	26,7	0	240031_at	AA994467	-36,6	1,14220607
35820_at	GM2A	26,5	0	207819_s_at	ABCB4	-36,2	0,81241439
222400_s_at	ADI1	26,4	0	1553891_at	KLF17	-35,5	0
205053_at	PRIM1	26,4	0,01763649	230861_at	DKFZP434L187	-35,3	0,34371104
205417_s_at	DAG1	26,4	0	229499_at	CAPN13	-35,2	0
227586_at	LOC124491	26,0	0	207764_s_at	HIPK3	-34,6	0
201459_at	RUVBL2	25,7	0	205051_s_at	KIT	-33,9	0,02177985
209600_s_at	ACOX1	25,5	0,02844238	230645_at	FRMD3	-33,9	0,34371104
228204_at	PSMB4	25,4	0	224444_s_at	C1orf97	-32,9	0,18873999
212191_x_at	RPL13	25,1	0	220657_at	KLHL11	-32,8	0
210319_x_at	MSX2	24,9	0,46529687	1555775_a_at	ZAR1	-32,7	0,02177985
218160_at	NDUFA8	24,5	0	230753_at	LOC197135	-31,3	0,02177985
214687_x_at	ALDOA	24,3	0	235736_at	Hs,178144	-31,2	0,34371104
217168_s_at	HERPUD1	24,1	0,01763649	1553086_at	C11orf40	-31,1	0,6041608
225799_at	MGC4677	23,9	0	237210_at	NFRKB	-31,0	0,03145261
44654_at	G6PC3	23,8	0	207623_at	ABCF2	-30,9	0,10846029
200658_s_at	PHB	23,8	0	214243_s_at	SERHL	-30,8	0,10846029
228155_at	C10orf58	23,7	0,01763649	214957_at	ACTL8	-30,7	0
223689_at	IGF2BP1	23,3	0	213326_at	VAMP1	-30,5	0,03145261
223075_s_at	C9orf58	22,7	0,18873999	205443_at	SNAPC1	-30,3	0,03145261
1560587_s_at	PRDX5	22,5	0	211915_s_at	LOC649679	-29,8	0
223206_s_at	NMRAL1	22,3	0	222361_at	LOC643224	-29,4	0
224469_s_at	C14orf151	22,2	0	220812_s_at	HHLA2	-29,4	0,05235772
224232_s_at	PX19	22,0	0	236491_at	BCL2L10	-29,0	0,6041608
208722_s_at	ANAPC5	22,0	0	1557085_at	TMEM122	-28,8	0,02177985
227492_at	OCLN	21,9	0	220108_at	GNA14	-28,2	0,13454973
230204_at	AU144114	21,9	0,02844238	230697_at	BBS5	-28,1	0,18873999
203113_s_at	EEF1D	21,7	0	221314_at	GDF9	-27,9	0,03145261
244567_at	Hs,125395	21,6	0,10846029	217047_s_at	FAM13A1	-27,8	0,13454973
226137_at	Hs,569686	21,6	0,01763649	204776_at	THBS4	-27,3	0,02177985
207332_s_at	TFRC	21,6	0	210162_s_at	NFATC1	-27,3	0,13454973
220289_s_at	AIM1L	21,6	0,81241439	218929_at	CARF	-27,2	0
223165_s_at	IHPK2	21,5	0,05235772	219686_at	STK32B	-27,2	0,6041608
217733_s_at	TMSB10	21,5	0	231472_at	FBXO15	-26,4	0
228077_at	MGC3207	21,2	0,01763649	236205_at	Hs,13188	-25,7	0,18873999
218140_x_at	SRPRB	21,1	0	1557700_at	POLH	-25,7	0,46529687
208837_at	TMED3	20,8	0	227337_at	ANKRD37	-25,6	0
201923_at	PRDX4	20,8	0,02844238	224800_at	WDFY1	-25,2	0
229120_s_at	CDC42SE1	20,7	0,01763649	224452_s_at	MGC12966	-25,0	0
202284_s_at	CDKN1A	20,6	1,14220607	230789_at	SUHW2	-25,0	0,10846029
221750_at	HMGCS1	20,6	0,13454973	205226_at	PDGFRL	-24,8	0,02177985
202289_s_at	TACC2	20,5	0,01763649	228007_at	C6orf204	-24,8	0,23707459
227792_at	LOC162073	20,3	0,05235772	205031_at	EFNB3	-24,6	0,46529687
229385_s_at	AI743780	20,3	0,05147485	228454_at	MLR2	-24,3	0,13454973
203234_at	UPP1	20,2	0	203855_at	WDR47	-24,3	0
218026_at	CCDC56	20,2	0	243943_x_at	C6orf52	-23,7	0
203190_at	NDUFS8	20,1	0	206010_at	HABP2	-23,7	0,03145261
202857_at	TMEM4	20,1	0	225655_at	UHRF1	-23,1	0,02177985
214501_s_at	H2AFY	20,0	0	241550_at	DPPA5	-23,1	0
225296_at	ZNF317	19,9	0	210495_x_at	FN1	-22,6	0
209429_x_at	EIF2B4	19,9	0	209392_at	ENPP2	-22,6	0
200862_at	DHCR24	19,9	0,02844238	219508_at	GCNT3	-22,6	0,0753266
205047_s_at	ASNS	19,8	0,13454973	242896_at	BF223302	-22,2	0
55081_at	MICAL-L1	19,8	0,02844238	207067_s_at	HDC	-22,2	0,10846029
204437_s_at	FOLR1	19,7	0,13454973	1553809_a_at	C9orf71	-22,1	0,10846029
212230_at	PPAP2B	19,6	0,81241439	228063_s_at	NAP1L5	-22,0	1,14220607
213011_s_at	TPI1	19,3	0	220399_at	FLJ22639	-22,0	0,81241439
225391_at	LOC93622	19,2	0,6041608	1555486_a_at	FLJ14213	-22,0	2,29260383
201121_s_at	PGRMC1	19,1	0,02844238	202464_s_at	PFKFB3	-21,9	0
201231_s_at	ENO1	19,1	0	231292_at	EID3	-21,6	0,10846029

211727_s_at	COX11	19,1	0,02844238
204416_x_at	APOC1	19,1	0,02844238
213187_x_at	FTL	19,1	0,02844238
228499_at	PFKFB4	19,1	0,01763649
203606_at	NDUFS6	19,0	0
223172_s_at	MTP18	18,9	0,01763649
202193_at	LIMK2	18,9	0,02844238
221245_s_at	C2orf31	18,8	0
206683_at	ZNF165	18,8	0,02844238
204868_at	ICT1	18,6	0,01763649
200897_s_at	PALLD	18,4	0
218526_s_at	RANGNRF	18,4	0
218035_s_at	FLJ20273	18,4	0
202454_s_at	ERBB3	18,3	3,15055114
225432_s_at	CSRP2BP	18,1	0
225168_at	FRMD4A	18,0	0,6041608
208308_s_at	GPI	17,8	0
219155_at	PITPNC1	17,7	0,02844238
228906_at	CXXC6	17,7	0,02844238
1552946_at	ZNF114	17,6	0,34371104
225342_at	AK3L1	17,6	0,34371104
214022_s_at	IFITM1	17,4	0,10846029
204975_at	EMP2	17,4	0,02844238
200645_at	GABARAP	17,3	0
206445_s_at	PRMT1	17,3	0
215091_s_at	GTF3A	17,3	0
200812_at	CCT7	17,2	0
202475_at	TMEM147	17,2	0
233049_x_at	STUB1	17,1	0
224573_at	MGC71993	17,1	0
210026_s_at	CARD10	17,1	0,02844238
217901_at	DSG2	17,0	0
209109_s_at	TSPAN6	16,8	0
225093_at	UTRN	16,8	0
218354_at	HSPC176	16,7	0
39729_at	PRDX2	16,5	0
213787_s_at	EBP	16,3	0,02844238
44146_at	GMEB2	16,2	0,0753266
204119_s_at	ADK	16,2	0
201416_at	SOX4	16,1	0
204306_s_at	CD151	16,1	0,05147485
200665_s_at	SPARC	16,1	0,34371104
200982_s_at	ANXA6	15,9	0,10846029
226905_at	FAM101B	15,8	0,18873999
218499_at	RP6-213H19,1	15,8	0,02844238
207760_s_at	NCOR2	15,8	0
209295_at	TNFRSF10B	15,8	0,13454973
226121_at	MGC23280	15,8	0,0753266
37117_at	ARHGAP8	15,7	0,10846029
213460_x_at	NSUN5C	15,7	0,01763649
209154_at	TAX1BP3	15,7	0
202961_s_at	ATP5J2	15,6	0
220864_s_at	NDUFA13	15,6	0
224588_at	XIST	15,6	0,46529687
219041_s_at	REPIN1	15,6	0
223222_at	SLC25A19	15,5	0
201740_at	NDUFS3	15,5	0
227897_at	RAP2B	15,5	0
202054_s_at	ALDH3A2	15,4	0,02844238
1555299_s_at	ERVWE1	15,4	0,02844238
202826_at	SPINT1	15,4	0,05147485
212501_at	CEBPB	15,4	2,29260383
200871_s_at	PSAP	15,3	0
209129_at	TRIP6	15,3	0,34371104
213670_x_at	NSUN5B	15,2	0,05147485
208908_s_at	CAST	15,2	0,10846029

243272_at	Hs,99308	-21,6	1,61463267
231051_at	SLC16A9	-21,5	0
238218_at	LOC648473	-21,5	0
231164_at	LOC440331	-21,4	0,03145261
219947_at	CLEC4A	-21,4	0,03145261
203355_s_at	PSD3	-21,2	0,02177985
226751_at	C2orf32	-21,1	0,0753266
237840_at	LOC388948	-21,0	0,23707459
209576_at	GNAI1	-20,7	0,03145261
237613_at	FOXR1	-20,6	0,02177985
203256_at	CDH3	-20,4	0,13454973
220310_at	TUBAL3	-20,4	0
225316_at	MFSD2	-20,2	0
218646_at	FLJ20534	-20,1	0
228875_at	C6orf189	-20,1	0,46529687
227997_at	IL17RD	-20,0	0,23707459
1554105_at	FAM11A	-19,9	0,05235772
231386_at	FLJ30851	-19,9	0,18873999
229744_at	SSFA2	-19,8	1,61463267
211596_s_at	LRIG1	-19,7	0,23707459
213452_at	ZNF184	-19,7	0,0753266
1561353_at	Hs,385477	-19,6	1,14220607
1558438_a_at	IGHG1	-19,2	0,02177985
210012_s_at	EWSR1	-19,0	0,18873999
232720_at	LRRN6C	-18,9	0,18873999
1562722_at	FLJ40296	-18,9	0,03145261
230288_at	AW418619	-18,7	0,34371104
226153_s_at	CNOT6L	-18,7	0
203705_s_at	FZD7	-18,7	0,10846029
37547_at	PTHB1	-18,6	0,81241439
225798_at	tcag7,981	-18,6	0,10846029
214481_at	HIST1H2AM	-18,5	3,15055114
1561673_at	Hs,434703	-18,3	0,13454973
213271_s_at	DOPEY1	-18,3	0,03145261
219518_s_at	ELL3	-18,2	0,6041608
205190_at	PLS1	-18,1	0
204071_s_at	TOPORS	-18,0	0
223846_at	AZI2	-17,9	0,6041608
236904_x_at	TECTA	-17,6	0
230847_at	T79870	-17,6	0,6041608
201174_s_at	TERF2IP	-17,6	0,18873999
206588_at	DAZL	-17,5	0
1554789_a_at	PDE8B	-17,5	0
230302_at	Hs,264606	-17,3	4,24774547
222921_s_at	HEY2	-17,3	0,46529687
222572_at	PPM2C	-17,0	0,03145261
207490_at	TUBA4	-16,9	0,46529687
213220_at	LOC92482	-16,9	0,02177985
218807_at	VAV3	-16,7	0,34371104
230854_at	BCAR4	-16,7	0,02177985
239715_at	LOC400927	-16,7	0,6041608
220167_s_at	TP53TG3	-16,6	0,03145261
227297_at	ITGA9	-16,5	1,61463267
205799_s_at	SLC3A1	-16,4	0,18873999
225930_at	NKIRAS1	-16,2	0
229888_at	C12orf60	-16,2	0,13454973
220019_s_at	ZNF224	-16,1	0,05235772
230542_at	ZNF597	-15,9	0,23707459
235948_at	FAM80A	-15,7	0,34371104
220534_at	TRIM48	-15,7	1,14220607
214505_s_at	FHL1	-15,7	0
1555396_s_at	LOC340602	-15,6	0,02177985
202981_x_at	SIAH1	-15,3	0,03145261
205854_at	TULP3	-15,2	0,0753266
243346_at	LMOD3	-15,1	0,10846029
239436_at	CHORDC1	-14,9	2,29260383

205133_s_at	HSPE1	15,2	0
203025_at	ARD1A	15,1	0
218249_at	ZDHHC6	15,0	0
226610_at	PRR6	15,0	0,05147485
201268_at	NME2	15,0	0
210512_s_at	VEGF	15,0	0,6041608

242421_at	Hs,613574	-14,7	0,10846029
1553713_a_at	RHEBL1	-14,6	0
1567623_at	ABLIM2	-14,6	0,81241439
209034_at	PNRC1	-14,6	0
204709_s_at	KIF23	-14,5	0,05235772
1562275_at	ADAMTS9	-14,5	0,18873999

Supplementary Table S5: The 200 most down-regulated in the EGA compared with the MII stage in mouse.

MII, metaphase II oocytes; EGA, embryonic genome activation stage.

Gene ID	Gene Name	Fold Change	q-value(%)
1427287_s_at	Itpr2	-139,3	0
1419474_a_at	Ehf	-129,1	0,578131308
1447830_s_at	Rgs2	-127,7	0
1416518_at	H1foo	-125,0	0
1434031_at	Zfp692	-108,2	0
1416961_at	Bub1b	-105,1	0
1420196_s_at	Tbc1d14	-94,4	0,091496987
1425869_a_at	Psen2	-92,9	0,039876518
1448499_a_at	Ephx2	-92,9	3,337876471
1416592_at	Glrx	-87,2	0
1428498_at	Rnf219	-83,6	0
1423086_at	Npc1	-82,3	0
1417483_at	Nfkbiz	-79,6	0
1428638_at	Efhc2	-75,0	0
1418925_at	Celsr1	-71,4	0
1438867_at	Gm1070	-69,1	0
1417904_at	Dclre1a	-67,1	0
1419041_at	Itfg1	-64,7	0
1427981_a_at	Csad	-63,0	0
1434158_at	Gmds	-62,7	0
1443478_at	C86695	-60,7	0
1438658_a_at	S1pr3	-58,9	0
1451191_at	Crabp2	-57,6	0
1451674_at	Slc12a5	-57,3	0
1452207_at	Cited2	-54,2	1,091655357
1430229_at	Gpr137c	-53,1	0
1434527_at	Nlrp4b	-52,6	0
1436884_x_at	Ewsr1	-52,2	0,197774187
1424060_at	Neil3	-51,0	0
1454678_s_at	A130022J15Rik	-50,9	0
1415917_at	Mthfd1	-50,5	0
1417046_at	Nprl2	-49,2	0
1431695_at	Rph3al	-48,3	0
1457976_at	2010002M12Rik	-47,5	0
1419511_at	Msh4	-47,3	0
1434494_at	Zar1	-46,9	0
1449925_at	Cxcr3	-46,9	0
1443679_at	2510003B16Rik	-46,6	0
1423723_s_at	Tardbp	-46,4	0
1419091_a_at	Anxa2	-45,5	2,048028413

1424821_at	LOC100046168 /// Ndfip1	-45,3	0
1434070_at	Jag1	-44,7	0
1438769_a_at	Thyn1	-44,5	0
1424076_at	Gdpd1	-43,6	0
1420931_at	Mapk8	-42,5	0
1428259_at	Pxdn	-42,2	0
1438445_at	Lrrc8e	-41,9	0
1424536_at	Oas1e	-41,4	0
1425049_at	Oas1h	-40,5	0
1456017_x_at	LOC666466 /// Obox2	-39,7	0
1432291_at	0610033M10Rik	-39,3	0
1447112_s_at	Cryl1	-39,0	0
1448999_at	Trappc5	-38,1	0
1417049_at	Rhd	-37,1	0
1415996_at	Txnip	-36,9	0
1423570_at	Abcg1	-35,5	0
1425567_a_at	Anxa5	-35,3	0
1422899_at	Slc6a20b	-35,3	0
1449059_a_at	Oxct1	-35,1	0
1417538_at	LOC100046775 /// Slc35a1	-34,7	0
1442081_at	Gm4632	-34,5	0
1417438_at	Rdh14	-34,4	0
1456183_at	Oog4	-34,2	0
1415722_a_at	Vta1	-33,9	0
1446845_at	Gm5065	-32,5	0
1427085_at	2810432D09Rik	-32,2	0
1436948_a_at	Fam70a	-32,2	0,039876518
1421133_at	LOC100047693 /// Pvrl3	-32,1	0
1455435_s_at	Chdh	-32,1	0
1435461_at	Magi3	-31,5	0
1431230_a_at	Btbd9 /// LOC100048111	-30,9	0
1419665_a_at	Nupr1	-30,8	3,337876471
1415806_at	Plat	-30,8	0
1415864_at	Bpgm	-30,3	0
1452621_at	Pcbd2	-30,1	0
1428384_at	D4Bwg0951e	-29,8	0
1429291_at	Psmd1	-29,5	0
1424114_s_at	Lamb1-1	-29,5	0
1415677_at	Dhrs1	-28,7	0
1460122_at	Tmem41b	-28,5	0
1439991_a_at	Pabpn1l	-28,1	0
1450915_at	Ap3b1	-27,7	0

1415947_at	Creg1	-27,6	0,197774187
1415698_at	Golm1	-27,6	0
1440556_at	Gm11985	-27,5	0
1433972_at	Camta1	-27,5	0
1449108_at	Fdx1	-27,0	0,136811798
1441945_s_at	Abhd14a	-26,9	0,022595242
1438077_at	Nlrp4a	-26,8	0
1428985_at	Ints12	-26,8	0
1452837_at	Lpin2	-26,7	0
1420903_at	St6galnac3	-26,6	0,022595242
1427031_s_at	Ccdc52	-26,5	0
1424042_at	Tmem5	-26,4	0
1423301_at	Copb1	-26,3	0
1427964_at	Cmtm8	-26,3	0
1450720_at	Acp1	-26,2	0
1428045_a_at	Elf2	-25,5	0,091496987
1431772_a_at	Itsn2	-25,5	0
1420174_s_at	Tax1bp1	-25,4	0
1446919_at	Zfp873	-25,4	0
1419716_a_at	Pou2f1	-25,3	0
1434830_at	Mxd1	-25,3	0
1448172_at	Mdh1	-25,0	0
1439721_at	Fbxw18	-24,7	0
1448317_at	Tmem128	-24,6	0,039876518
1437370_at	Sgol2	-24,2	0
1423786_at	8430410A17Rik	-24,2	0
1424299_at	Oma1	-24,0	0,022595242
1452671_s_at	Lman1	-24,0	0
1416032_at	Tmem109	-24,0	0
1429265_a_at	Rnf130	-24,0	0
1418815_at	Cdh2	-24,0	0
1416509_at	Tm9sf3	-24,0	0
1441295_at	Lman2l	-23,9	0
1457084_at	Nlrp9b	-23,9	0
1438971_x_at	Ube2h	-23,8	0
1437307_at	Senp8	-23,7	0
1433579_at	Tmem30b	-23,3	0
1445723_at	Plcl1	-23,2	0
1448319_at	Akr1b3	-23,2	0
1436419_a_at	1700097N02Rik	-23,0	0
1459252_at	Gm4981	-23,0	0
1415840_at	Elovl5	-22,9	0
1457028_at	A430033K04Rik	-22,9	0,022595242
1418437_a_at	Mlx	-22,9	0
1448120_at	Gdf9	-22,8	0
1449130_at	Cd1d1	-22,6	0

1420808_at	Gm6768 /// Ncoa4	-22,6	0
1456721_at	Thsd7a	-22,6	0
1452828_at	Fbxo21	-22,5	0
1448398_s_at	Rpl22	-22,5	0
1418539_a_at	Ptpre	-22,5	0,091496987
1455823_at	Bbs4	-22,3	0
1434963_at	Supt3h	-22,3	1,091655357
1423297_at	Add3	-22,2	0
1433940_at	Spag7	-22,1	0
1451177_at	Dnajb4	-22,1	0
1417527_at	Ap3m2	-22,0	0
1447173_at	Lrrc31	-21,9	0
1418109_at	Gspt2	-21,9	0
1436899_at	Zufsp	-21,9	0
1436588_at	Rttn	-21,8	0
1452199_at	Tmem209	-21,8	0
1427163_at	Ubr2	-21,7	0
1415791_at	Rnf34	-21,7	0
1453155_at	Tmem50a	-21,7	0
1436242_a_at	Cklf	-21,7	0
1417245_at	Gpr180	-21,6	0
1429506_at	LOC634379 /// Nkd1	-21,4	0
1453090_x_at	LOC666466 /// Obox1 /// Obox2	-21,4	0
1428023_at	3110009E18Rik	-21,4	0
1424138_at	Rhbdf1	-21,0	0,039876518
1438653_x_at	Atxn10	-20,9	3,337876471
1460257_a_at	Gm2382 /// Mthfs	-20,9	0
1439046_at	Ccdc55	-20,8	0,039876518
1460123_at	Gpr1	-20,8	0
1416226_at	Arpc1b	-20,6	0
1428905_at	Rraga	-20,6	0,091496987
1448470_at	Fbp1	-20,6	0
1456185_at	Nlrp9a	-20,6	0
1429679_at	Lrrc17	-20,4	0
1441534_at	C86753	-20,4	0
1451391_at	2700050L05Rik	-20,3	0
1452087_at	Epsti1	-20,3	0
1416846_a_at	Pdzrn3	-20,2	0
1455066_s_at	Mia3	-20,2	0
1454782_at	Bai3	-20,1	0
1415679_at	Psenen	-19,8	0
1451302_at	1110012L19Rik	-19,8	0
1458438_at	Ccdc122	-19,7	0
1416270_at	Polr2g	-19,6	0
1428243_at	1700021K19Rik	-19,6	0

1423568_at	Psma7	-19,6	0,197774187
1453700_s_at	4933403O03Rik /// Gm4981	-19,5	0,312577673
1424442_a_at	Pja2	-19,5	0,136811798
1437755_at	Slc5a12	-19,5	0
1428682_at	Zc3h6	-19,5	0
1424080_at	Dcps	-19,3	0
1430735_at	Patl2	-19,3	0
1454262_at	Fbxw27	-19,2	0
1434753_at	Nfrkb	-19,2	0
1424218_a_at	Creb3l4	-19,2	0
1424686_at	Heatr6	-19,2	0
1448509_at	Fam107b	-19,1	0
1451283_at	Fam114a2	-19,0	0
1448910_at	Pecr	-19,0	0
1459897_a_at	Sbsn	-19,0	3,337876471
1427957_at	9530008L14Rik	-18,8	0
1424370_s_at	Psmf1	-18,7	0
1419630_a_at	Trim11	-18,7	0
1426417_at	Yipf4	-18,3	0
1451756_at	Flt1	-18,3	0
1436181_at	Asap2	-18,3	0
1449329_at	Zfp235	-18,2	0
1448971_at	2410022L05Rik	-18,2	0
1448445_at	Acp6	-18,2	0
1451924_a_at	Edn1	-18,1	0
1415897_a_at	Mgst1	-18,1	3,337876471
1460353_at	Tmem48	-18,1	0

Supplementary Table S6: The 200 most up- and down-regulated in the BL compared with the MII stage in mouse.
MII, metaphase II oocytes; BL, blastocyts.

Gene ID	Gene Name	Fold Change	q-value(%)	Gene ID	Gene Name	Fold Change	q-value(%)
1417116_at	Slc6a8	269,5	0	1455851_at	Bmp5	-160,0	0
1448263_a_at	Cndp2	174,8	0	1423226_at	Ms4a1	-108,4	0
1417346_at	Pycard	137,8	0	1432291_at	0610033M10Rik	-102,2	0
1427893_a_at	Pmvk	78,6	0	1455823_at	Bbs4	-83,2	0
1450107_a_at	Renbp	55,2	0	1449824_at	Prg4	-75,9	0
1452049_at	Rpl7l1	49,2	0	1455914_at	AI987944	-75,0	0
1436816_at	Nup133	43,1	0	1435667_at	Rims1	-74,1	0
1416278_a_at	Atp5o /// LOC100047429	42,4	0	1440856_at	Mapk8	-73,3	0
1416604_at	Cyc1	39,7	0	1435261_at	Tmtc1	-70,5	0
1447934_at	9630033F20Rik	38,9	0	1454782_at	Bai3	-70,1	0
1449137_at	Pdha1	36,7	0	1438297_at	AA545190	-69,1	0
1419509_a_at	Nagk	35,4	0	1426708_at	Antxr2	-66,7	0
1438000_x_at	Zfp622	34,9	0	1428682_at	Zc3h6	-60,1	0
1423724_at	Zwint	34,4	0	1448557_at	Fam13c	-59,6	0
1452880_at	Znhit3	34,3	0	1422021_at	Spry4	-58,0	0
1416789_at	Idh3g	32,7	0	1439167_at	Pecr	-57,8	0
1416146_at	Hspa4	32,4	0	1449925_at	Cxcr3	-55,9	0
1451336_at	Lgals4	31,5	0	1429303_at	Klf17	-55,7	0
1416478_a_at	Mdh2	31,1	0	1451191_at	Crabp2	-50,5	0
1415681_at	Mrpl43	30,2	0	1427278_at	Clip4	-50,1	0
1449061_a_at	Prim1	29,8	0	1430200_at	4930579D07Rik /// LOC100044513	-49,0	0
1451401_a_at	Mul1	29,8	0	1448136_at	Enpp2	-47,7	0
1424313_a_at	Ndufs7	29,6	0	1452088_at	Zbed3	-45,3	0
1452048_at	Mrpl12	29,4	0	1460081_at	Syt7	-44,4	0
1453120_at	Tmx4	29,0	0	1440782_at	Skap1	-44,2	0
1424877_a_at	Alad /// LOC100046072	28,1	0	1434830_at	Mxd1	-44,1	0
1424200_s_at	Seh1l	27,4	0	1439059_at	Fam199x	-42,7	0
1434702_at	Ddrgk1	27,3	0	1441122_at	LOC639236	-42,4	0
1451248_at	Prmt7	27,0	0	1417129_a_at	Meis2	-41,4	0
1453207_at	2900053A13Rik	26,9	0	1421065_at	Jak2	-41,3	0
1452680_at	Snrpd2	26,9	0	1420851_at	Pard6g	-39,0	0
1434923_at	Cox19	26,9	0	1424631_a_at	Ighg	-38,9	0
1424628_a_at	Ndufv3	26,8	0	1453166_at	Ccdc109a	-38,8	0
1448314_at	Cdk1	26,8	0	1454962_at	Spire1	-38,8	0
1452628_at	Bag5 /// LOC100047042	26,2	0	1430897_at	4931428L18Rik	-38,7	0
1438159_x_at	Ndufv2	26,1	0	1418659_at	Clock	-38,6	0
1455333_at	Tns3	25,9	0	1421240_at	Ern1	-38,6	0
1437220_x_at	Psmd13	25,9	0	1450923_at	Tgfb2	-38,2	0
1455106_a_at	Ckb	25,5	0	1419542_at	Dazl	-38,2	0
1423049_a_at	Tpm1	24,9	0	1421594_a_at	Sytl2	-38,0	0
1424001_at	Mki67ip	24,8	0	1437112_at	Pld1	-37,7	0
1449004_at	Mrpl46	24,2	0	1460255_at	Tnfsf13b	-37,5	0
1422517_a_at	Znrd1	24,0	0	1445473_at	Diras2	-37,1	0
1435995_at	Mrpl22	22,8	0	1417644_at	Sspn	-36,6	0
1418794_at	Cds2	22,7	0	1456741_s_at	Gpm6a	-36,5	0
1415676_a_at	Psmb5	22,2	0	1429759_at	Rps6ka6	-35,8	0
1452954_at	Ube2c	22,0	0	1456033_at	Tbx4	-35,5	0
1416020_a_at	Atp5g1	22,0	0	1421514_a_at	LOC100047360 /// Scml2	-34,9	0
1423991_at	Nop14	22,0	0	1419434_at	Slc2a10	-34,5	0
1428322_a_at	Ndufb10	21,9	0	1440476_at	D6Ertd474e	-34,4	0
1417105_at	Trappc2l	21,6	0	1435660_at	LOC664787	-33,6	0
1418124_at	Tmem85	21,5	0	1451476_at	Zdhhc8	-33,2	0
1451015_at	Tkt	21,4	0	1443478_at	C86695	-32,8	0
1428766_at	Rnmtl1	21,4	0	1417331_a_at	Arl6	-32,4	0
1448882_at	Tmem93	21,1	0	1452604_at	Stard13	-32,1	0
1416608_a_at	BC004004	21,0	0	1433919_at	Asb4	-32,1	0
1430500_s_at	Mtx2	20,9	0	1434252_at	Tmcc3	-31,9	0
1450818_a_at	Ndufa7	20,7	0	1429475_at	LOC677224 /// Ubash3b	-31,9	0
1424033_at	Sfrs7	20,7	0	1419231_s_at	Krt12	-31,2	0
1416438_at	Puf60	20,5	0	1425846_a_at	Caln1	-31,2	0
1422912_at	Bmp4	20,4	0	1437732_at	Omt2a	-30,7	0
1417448_at	1810008A18Rik	20,2	0	1425776_a_at	C87436	-30,4	0
1451521_x_at	Eif4h	20,1	0	1456425_at	9230115E21Rik	-30,3	0
1448802_at	Nufip1	20,0	0	1424822_at	Slain1	-29,7	0
1451002_at	Aco2	19,9	0	1419403_at	BC017612	-29,7	0
1423670_a_at	Srpr	19,3	0	1457401_at	Dnahc9	-29,0	0
1438317_a_at	Endog	19,0	0	1448556_at	Prlr	-28,1	0
1434113_a_at	Rexo4	18,9	0	1428899_at	Tmem182	-28,1	0
1428658_at	Pin4	18,8	0	1460606_at	Hsd17b13	-27,9	0
1416627_at	Spint1	18,8	0	1437312_at	Bmpr1b	-27,8	0
1436302_at	Slc10a7	18,7	0	1435992_at	Iqca	-27,7	0

1450919_at	Mpp1	18,6	0
1424019_at	Nop2	18,4	0
1419287_at	Tmem208	18,3	0
1440826_s_at	2610002I17Rik	18,1	0
1424500_at	Utp6	18,1	0
1416276_a_at	Rps4x	18,0	0
1453097_a_at	Ubtf	18,0	0
1453052_at	Tmem192	17,9	0
1420000_s_at	Igbp1	17,8	0
1416240_at	Psmb7	17,8	0
1433882_at	Cnot10	17,5	0
1422128_at	Rpl14	17,0	0
1434543_a_at	Bola2	16,9	0
1427988_s_at	Safb2	16,7	0
1424776_a_at	Slc25a28	16,5	0
1416417_a_at	Ndufb7	16,5	0
1415967_at	Ndufv1	16,3	0
1416667_at	Ebp	16,1	0
1450657_at	Ppie	16,1	0
1426674_at	Eif3b	15,7	0
1435006_s_at	Abcb7	15,7	0
1425196_a_at	Hint2	15,6	0
1416368_at	Gsta4	15,4	0
1416070_a_at	Ddx18	15,3	0
1452616_s_at	Ssbp1	15,3	0
1451745_a_at	Znhit1	15,2	0
1416327_at	Ufc1	15,2	0
1450638_at	Pdcd5	15,0	0
1438164_x_at	Flot2	14,9	0
1451272_a_at	Ube2f	14,9	0
1418101_a_at	Rtn3	14,7	0
1424651_at	Acsf3	14,7	0
1426846_at	Cenpt	14,6	0
1416663_at	Ndufa9	14,6	0
1416773_at	Wee1	14,5	0
1424538_at	Ubl4	14,4	0
1425993_a_at	Hsph1	14,4	0
1448524_s_at	Ssr4	14,3	0
1429485_a_at	Utp11l	14,3	0
1452128_a_at	Brcc3	14,1	0
1420623_x_at	Hspa8	14,1	0
1424058_at	Prrc1	14,0	0
1428898_at	Mon1a	14,0	0
1456727_a_at	Csnk1d	14,0	0
1454856_x_at	Rpl35	13,9	0
1452186_at	Rbm5	13,8	0
1448234_at	Dnajb6	13,6	0
1426902_at	Coq6	13,6	0
1423741_at	Rbm10	13,5	0
1438656_x_at	Timm17b	13,5	0
1417737_at	Mrps31	13,4	0
1438835_a_at	Eftud2	13,4	0
1436722_a_at	Actb	13,3	0
1450705_at	Rdbp	13,3	0
1456055_x_at	Pold1	13,3	0
1452586_at	Anapc13	13,2	0
1437414_at	Zfp217	13,1	0
1448604_at	Uck2	13,0	0
1450968_at	Uqcrfs1	12,9	0
1451081_a_at	Tcf25	12,9	0
1456117_at	Rrp1b	12,8	0
1418965_at	Nosip	12,8	0
1416243_a_at	Gm10269 /// Gm4342 /// Rpl35	12,8	0
1417698_at	Gtf2f1	12,7	0
1420616_at	Ash2l	12,7	0
1420830_x_at	Ywhaq	12,7	0
1450865_s_at	Mrps24	12,6	0
1439764_s_at	Igf2bp2	12,5	0
1424451_at	Acaa1b	12,4	0
1452919_a_at	Pgp	12,4	0
1424364_a_at	Uqcr10	12,4	0
1433611_s_at	Bud31	12,3	0
1447724_x_at	Opa3	12,3	0
1423080_at	Tomm20	12,3	0

1440215_at	Gm11818	-27,4	0
1455321_at	Ddhd1	-27,1	0
1456299_at	E330012B07Rik	-26,9	0
1420715_a_at	Pparg	-26,9	0
1448269_a_at	Klhl13	-26,6	0
1423328_at	Gdap1	-26,5	0
1447786_at	Cyth1	-26,2	0
1434834_at	Socs7	-26,1	0
1434440_at	Gnai1	-26,0	0
1439480_at	Gm10857	-25,9	0
1419511_at	Msh4	-25,9	0
1419110_at	Riok1	-25,8	0
1431356_at	6430710C18Rik	-25,8	0
1420928_at	St6gal1	-25,8	0
1424218_a_at	Creb3l4	-25,8	0
1418685_at	Tirap	-25,7	0
1459044_at	AU015558	-25,7	0
1455856_at	Gm1965	-25,6	0
1450105_at	Adam10	-25,4	0
1427359_at	Jhdm1d	-25,1	0
1427252_at	Dmrtb1	-24,7	0
1460611_at	C87499	-24,6	0
1435385_at	Tshz2	-24,6	0
1429679_at	Lrrc17	-24,5	0
1417806_at	Popdc2	-24,5	0
1444232_at	Prkg1	-24,4	0
1451535_at	Il31ra	-24,2	0
1431772_a_at	Itsn2	-24,2	0
1417179_at	Tspan5	-24,0	0
1416650_at	Rfpl4	-23,9	0
1460665_a_at	Cnot7	-23,9	0
1434592_at	Slc16a10	-23,8	0
1427253_s_at	Suz12	-23,8	0
1428696_at	Rftn1	-23,8	0
1434228_at	Pdp1	-23,8	0
1419354_at	Klf7	-23,5	0
1456221_at	Gm10632	-23,5	0
1434469_at	Otud4	-23,4	0
1455266_at	Kif5c	-23,2	0
1417336_a_at	Sytl4	-23,1	0
1417677_at	Opn3	-23,1	0
1429114_at	Sestd1	-22,9	0
1436861_at	Il7	-22,5	0
1436721_x_at	Oog3	-22,5	0
1419629_at	Mesp2	-22,4	0
1425099_a_at	Arntl	-22,4	0
1425190_a_at	Phospho2	-22,3	0
1441128_at	2900016J10Rik	-22,1	0
1418872_at	Abcb1b	-22,0	0
1428306_at	Ddit4	-21,9	0
1422831_at	Fbn2	-21,8	0
1455872_at	Fam167a	-21,6	0
1436805_at	Ubash3b	-21,6	0
1456847_at	Cd160	-21,6	0
1437469_at	Zfp750	-21,4	0
1454917_at	Alg10b	-21,4	0
1429110_a_at	Nsun4	-21,3	0
1427956_at	Pcgf1	-21,2	0
1442013_at	Ccdc79	-21,0	0
1433865_at	E330010L02Rik /// E330016L19Rik /// Gm2101 /// Gm2309 /// Gm7479	-20,8	0
1423371_at	Pole4	-20,6	0
1450730_at	Hs2st1	-20,6	0
1416542_at	Phf1	-20,5	0
1438466_at	Dnahc7b	-20,4	0
1448733_at	Bmi1	-20,3	0
1417977_at	Eif4e3	-20,3	0
1456684_at	Tmem74	-20,3	0
1454867_at	Mn1	-20,2	0
1450069_a_at	Celf2	-20,2	0
1434070_at	Jag1	-20,1	0
1417226_at	Fbxw4	-20,1	0
1450129_a_at	Socs6	-19,9	0
1425481_at	Cnot6l	-19,8	0
1420455_at	Gcm2	-19,8	0

1454709_at	Tmem64	12,3	0
1417873_at	Pwp1	12,3	0
1451421_a_at	Rogdi	12,2	0
1416256_a_at	Tubb5	12,1	0
1417390_at	Gpn1	12,1	0
1452169_a_at	Dgkz	12,1	0
1437301_a_at	Dvl1	12,0	0
1454214_a_at	Znhit6	11,9	0
1451083_s_at	Aars	11,9	0
1424696_at	Gpr89	11,9	0
1425616_a_at	Ccdc23	11,9	0
1427966_at	Fam105b	11,9	0
1434157_at	Txlna	11,8	0
1428004_at	Snrnp25	11,8	0
1416472_at	Syap1	11,7	0
1436803_a_at	Ndufb9	11,7	0
1416366_at	Ndufc2	11,7	0
1424109_a_at	Glo1	11,6	0
1426644_at	Tbc1d20	11,5	0
1439017_x_at	Adipor1	11,4	0
1423616_at	Tarbp2	11,2	0
1452446_a_at	Tmub2	11,2	0
1422476_at	Ifi30	11,2	0
1423889_at	Gm5617	11,1	0
1427909_at	2410015M20Rik	11,1	0
1420013_s_at	Lss	11,1	0
1427901_at	Mrps18c	11,1	0
1416059_at	LOC100048726 /// Sec23b	11,0	0
1417511_at	Lyar	11,0	0
1417321_at	Zcchc7	11,0	0
1450948_a_at	Mrpl1	11,0	0
1451736_a_at	Map2k7	10,9	0
1428589_at	Mrpl41	10,8	0
1423264_at	Bop1	10,8	0
1424347_at	Ppp6c	10,7	0
1416679_at	Abcd3	10,5	0
1428075_at	Gm3244 /// Ndufb4	10,5	0
1426453_at	Pitrm1	10,4	0
1426257_a_at	Sars	10,3	0
1426256_at	Timm17a	10,2	0
1428381_a_at	Ppdpf	10,2	0
1418578_at	Dgka	10,2	0
1452454_at	Sdad1	10,2	0
1421945_a_at	Rpf2	10,1	0
1415980_at	Atp5g2	10,1	0
1429453_a_at	Mrpl55	10,1	0
1424047_at	Dera	10,0	0
1423801_a_at	Aprt	10,0	0
1448621_a_at	Smpd1	10,0	0
1415683_at	Nmt1	9,9	0
1448589_at	Ndufb5	9,9	0
1438644_x_at	Commd9	9,8	0
1435243_at	Zfp746	9,8	0
1434119_at	D2Wsu81e	9,8	0
1455832_a_at	Umps	9,8	0

1420860_at	Itga9	-19,6	0
1457435_x_at	Myom2	-19,6	0
1417926_at	Ncapg2	-19,5	0
1437415_at	4933427D06Rik	-19,3	0
1423186_at	Tiam2	-19,2	0
1455710_x_at	Mtcp1	-19,2	0
1428789_at	Ralgps2	-19,2	0
1415677_at	Dhrs1	-19,1	0
1459089_at	Gm11756 /// Gm11757 /// Gm13871	-19,0	0
1455865_at	Insm1	-19,0	0
1435749_at	Gda	-18,9	0
1439571_at	E230008J23Rik	-18,9	0
1428243_at	1700021K19Rik	-18,9	0
1415731_at	Angel2	-18,8	0
1460220_a_at	Csf1	-18,7	0
1422824_s_at	Eps8	-18,6	0
1419588_at	Spag1	-18,6	0
1451221_at	BC018507	-18,6	0
1451510_s_at	Olah	-18,5	0
1415890_at	Papss1	-18,3	0
1417676_a_at	Ptpro	-18,3	0
1439945_at	Zfp449	-18,3	0
1437299_at	Nlrp4g	-18,3	0
1424029_at	Tspyl4	-18,2	0
1442659_at	Pcdh9	-18,1	0
1431564_at	Bcar3	-18,0	0
1453247_at	Zfp618	-18,0	0
1416125_at	Fkbp5	-18,0	0
1423754_at	Ifitm3	-17,9	0
1436314_at	Scyl2	-17,7	0
1424054_at	Btbd2	-17,7	0
1418749_at	Psd3	-17,5	0
1417430_at	Cdr2	-17,5	0
1444712_at	Hsf5	-17,3	0
1442544_at	Igh-6	-17,3	0
1433751_at	Slc39a10	-17,2	0
1456183_at	Oog4	-17,1	0
1427023_at	Phyhipl	-17,1	0
1421881_a_at	Elavl2	-17,0	0
1416981_at	Foxo1	-17,0	0
1416267_at	Scoc	-16,9	0
1415904_at	Lpl	-16,9	0
1423470_at	Ptbp2	-16,8	0
1417555_at	Atad1	-16,8	0
1428260_at	Atl1	-16,8	0
1426523_a_at	Gnpda2	-16,5	0
1425330_a_at	Gm7895 /// Ppm1b	-16,5	0
1417483_at	Nfkbiz	-16,4	0
1433894_at	Jazf1	-16,4	0
1428353_at	Foxk2	-16,3	0
1452087_at	Epsti1	-16,2	0
1448892_at	Dock7	-16,1	0
1436518_at	Usp46	-16,1	0
1457179_at	Nlrp4e	-16,1	0
1424131_at	Col6a3	-16,0	0

Supplementary Table S7: The 200 most up- and the 3 down-regulated in the BL compared with the EGA stage in mouse.
EGA, embryonic genome activation stage; BL, blastocyts.

Gene ID	Gene Name	Fold Change	q-value(%)
1422075_at	Cdx2	1103,3	0,2799642
1420647_a_at	Krt8	585,8	0,2799642
1416225_at	Adh1	369,6	0,3384012
1456642_x_at	S100a10	363,7	0,3384012
1416790_a_at	Gm5806 /// Gm9855 /// Tdg	300,5	0,2206964
1456174_x_at	Ndrg1	278,5	0,3727146
1419091_a_at	Anxa2	275,0	0,2799642
1428146_s_at	Acaa2	266,3	0,157687
1434909_at	Rragd	251,7	0,3384012
1415947_at	Creg1	237,6	0,1013274
1417185_at	Ly6a	214,3	1,7053108
1435176_a_at	Id2	194,8	0,3727146
1416023_at	Fabp3	170,0	0,2092861
1416531_at	Gsto1	167,7	0,1013274
1429377_at	2410004A20Rik	165,9	0,2206964
1455439_a_at	Lgals1	157,6	0
1448894_at	Akr1b8	147,5	0,2206964
1424572_a_at	H2afy	146,3	0,2799642
1417125_at	Ahcy	146,2	0,1013274
1450138_a_at	Serpinb6a	143,9	0,1013274
1420000_s_at	Igbp1	141,1	0
1422128_at	Rpl14	134,4	0
1449024_a_at	Hexa	134,1	0,2799642
1417777_at	Ptgr1	117,0	0,2799642
1418133_at	Bcl3	114,6	0,3245931
1450989_at	Tdgf1	112,8	1,2786576
1417116_at	Slc6a8	112,2	0
1428464_at	Ndufa3	108,2	0,157687
1417285_a_at	Ndufa5	105,4	0,157687
1451112_s_at	Dap	100,6	0,2799642
1448393_at	Cldn7	90,8	0,3245931
1422851_at	Hmga2	90,0	0,2206964
1455972_x_at	Hadh	88,0	0,157687
1438653_x_at	Atxn10	86,2	0,2799642
1447604_at	BC053393	85,2	0,2206964
1429265_a_at	Rnf130	84,1	0,1013274
1416119_at	Txn1	79,5	0,3727146
1427133_s_at	Lrp2	71,2	0,2799642
1429311_at	Ube2q1	70,2	0
1423692_at	Ndufa8	69,4	0
1424353_at	Lrpprc	67,9	0,157687
1418711_at	Pdgfa	67,8	0,2206964
1448956_at	Stard10	65,8	0,157687
1454678_s_at	A130022J15Rik	65,4	0
1455333_at	Tns3	65,2	0
1433509_s_at	Reep1	63,2	0,3384012
1417349_at	Pldn	60,2	0,2799642
1433559_at	Slc45a4	59,6	0,1013274
1419812_s_at	Ccdc56	58,9	0,2092861
1448573_a_at	Ceacam10	58,3	1,7053108
1416592_at	Glrx	56,6	0,3727146
1423716_s_at	Atp5d	56,0	0
1419289_a_at	Syngr1	55,2	0,1013274
1426715_s_at	Slc46a1	53,9	0,3245931
1436349_at	2700094K13Rik	53,9	0,2092861
1453572_a_at	Plp2	52,8	0,1013274
1428181_at	Etfb	52,4	0,1013274
1425464_at	Gata6	52,4	0,157687
1423596_at	Nek6	50,6	0
1433504_at	Pygb	50,5	0,157687
1437723_s_at	Derl1	50,1	0,2799642
1424562_a_at	Slc25a4	49,5	0,1013274
1417052_at	Psmb3	49,4	0,2206964
1433467_at	Slc7a6	49,1	0,3384012
1433883_at	Tpm4	48,8	0,3245931
1426981_at	Pcsk6	47,9	0,2799642
1424345_s_at	Ube2m	47,4	0,6644655
1434067_at	AI662270	47,2	0,5161509

Gene ID	Gene Name	Fold Change	q-value(%)
1427479_at	BB287469 /// Eif1a /// Gm2022 /// Gm4027 /// Gm5039 /// Gm5662 /// Gm5788 /// Gm6804 /// LOC641136	-87,6	4,0435592
1460065_at	AA763515	-21,7	4,0435592
1441275_at	Kbtbd8	-6,5	4,0435592

1424184_at	Acadvl	46,9	0,2799642
1423568_at	Psma7	46,4	0,1013274
1437454_a_at	Tmx2	45,7	0,2799642
1449575_a_at	Gstp1	45,5	0,1013274
1429701_at	Mageb16	45,4	0,2092861
1451418_a_at	Spsb4	45,2	0
1436884_x_at	Ewsr1	44,8	0,2799642
1422836_at	Mbnl3	44,7	0,8103388
1416257_at	Capn2	44,6	0,3727146
1417443_at	Fam151a	44,4	0,2799642
1436902_x_at	Gm9844 /// Tmsb10	43,0	0,2206964
1416009_at	Tspan3	42,8	0,6644655
1415834_at	Dusp6	42,6	0,3245931
1415891_at	Suclg1	42,4	0,1013274
1455286_at	Btbd1	42,3	0,3384012
1415779_s_at	Actg1	42,1	0,2799642
1438610_a_at	Cryz	41,6	0,2799642
1433519_at	Nucks1	41,2	0,2799642
1452446_a_at	Tmub2	41,1	0
1455019_x_at	Ckap4	40,9	0
1424877_a_at	Alad /// LOC100046072	40,8	0
1418091_at	Tcfcp2l1	40,0	0,2799642
1450878_at	Sri	39,6	0
1436722_a_at	Actb	39,4	0
1452111_at	Mrps35	39,0	0
1435493_at	Dsp	38,8	0,3384012
1426690_a_at	Srebf1	38,6	0
1416271_at	Perp	38,5	0,3384012
1422476_at	Ifi30	38,3	0
1428373_at	Ip6k2	38,0	0,2799642
1455391_at	Rad23a	37,7	0,3245931
1436640_x_at	Agpat4	37,7	0
1450668_s_at	Hspe1	37,2	0,1013274
1422567_at	Fam129a	37,0	0,2799642
1452716_at	5730469M10Rik	36,9	0,6644655
1423588_at	Arpc4	36,8	0,157687
1452207_at	Cited2	36,3	0,3727146
1424038_a_at	2310044H10Rik	36,1	0
1424991_s_at	Tyms /// Tyms-ps	36,1	0
1419041_at	Itfg1	34,9	0
1458218_s_at	Pde7a	34,8	0,3727146
1417737_at	Mrps31	34,6	0
1418417_at	Msc	34,5	0,2206964
1434963_at	Supt3h	34,1	2,1743407
1424048_a_at	Cyb5r1	33,9	0,2206964
1451247_at	Mfsd1	33,8	0,2206964
1418038_s_at	Dusp19	33,6	0
1450721_at	Acp1	33,3	0
1421996_at	Tcfap2a	33,0	0,8103388
1417392_a_at	Slc7a7	33,0	0,2799642
1416449_x_at	Stxbp2	32,9	0,2206964
1415759_a_at	Hbxip	32,8	0
1435450_at	Cpne3	32,5	0,2799642
1417240_at	Zyx	32,4	0,8103388
1430596_s_at	Vgll3	32,2	0,8103388
1436609_a_at	Lrpap1	32,2	0,2206964
1437624_x_at	Nudt16l1	32,2	0,1013274
1416509_at	Tm9sf3	32,2	0,2799642
1434923_at	Cox19	31,9	0
1452740_at	Myh10	31,9	0,2799642
1424400_a_at	Aldh1l1 /// LOC100047937	31,9	0,2799642
1416604_at	Cyc1	31,9	0
1434031_at	Zfp692	31,8	0,3727146
1437423_a_at	Sra1	31,2	0
1428075_at	Gm3244 /// Ndufb4	31,1	0
1453001_at	Rnaseh2b	30,9	0,2092861
1423960_at	Lpcat3	30,9	0,3384012
1428314_at	Pcnp	30,9	0,2799642
1418347_at	Ccdc22	30,9	0
1441980_at	C030007I09Rik	30,5	0,5161509
1428283_at	Cyp2s1	30,5	0,3727146
1454641_at	Cggbp1	30,4	0,3384012
1448563_at	Phb	30,4	0
1450627_at	Ank	30,0	0,2206964
1453207_at	2900053A13Rik	30,0	0
1448353_x_at	Rpn1	29,8	0

1423086_at	Npc1	29,8	0
1422178_a_at	Rab17	29,6	0,3245931
1416368_at	Gsta4	29,6	0
1448485_at	Ggt1	29,0	0,2799642
1438672_at	Parvb	29,0	0,6644655
1437992_x_at	Gja1	28,9	0,2799642
1455680_at	9630025H16Rik /// Kpna4	28,8	0,6644655
1417177_at	Galk1	28,8	0,2799642
1452225_at	2010106G01Rik	28,8	0,2799642
1416948_at	Mrpl23	28,6	0,1013274
1449450_at	Ptges	28,6	0,2206964
1430176_at	Kbtbd13	28,4	0,6644655
1456390_at	Ppp2ca	28,3	0,3245931
1425788_a_at	Echdc2	28,2	0,6644655
1426001_at	Eomes	28,1	0,2799642
1428546_at	Syncrip	27,9	0,3245931
1449412_at	1810046J19Rik	27,7	0,3384012
1460235_at	Scarb2	27,7	0,2092861
1456377_x_at	Limd2	27,6	0,3727146
1419181_at	Zfp326	27,1	1,2786576
1425680_a_at	Btrc	26,9	0
1438835_a_at	Eftud2	26,8	0
1433489_s_at	Fgfr2	26,8	0,5161509
1448491_at	Ech1	26,7	0,1013274
1422608_at	Arpp19 /// LOC632823	26,5	0
1455435_s_at	Chdh	26,5	0,8103388
1415738_at	Txndc12	26,4	0,2092861
1448886_at	Gata3	26,3	0,2799642
1423419_at	Lig3	26,3	0
1456439_x_at	Mical1	26,3	0
1417364_at	Eef1g /// LOC100047986	26,3	0,157687
1460592_at	Epb4.1l1	26,2	1,2786576
1426400_a_at	Capns1	26,1	0
1428619_at	Fam36a	25,8	0,3727146
1424293_s_at	Tmem55a	25,8	0,2206964
1448118_a_at	Ctsd	25,8	0,2799642
1448372_a_at	Cnpy2	25,8	0,157687
1445940_at	D4Ertd298e	25,5	0,2799642
1424344_s_at	Eif1a	25,5	0,2799642
1435140_at	Ide	25,3	0,157687
1437223_s_at	Xbp1	25,3	0,3384012
1415916_a_at	Mthfd1	25,3	0
1424442_a_at	Pja2	25,2	0,1013274
1451512_s_at	Hibch	25,0	0,2799642
1416142_at	Rps6	24,9	0,2799642
1423625_a_at	Dnajc19	24,7	0,3245931
1417026_at	Pfdn1	24,6	0
1422433_s_at	Idh1	24,5	0
1448967_at	Nipsnap3b	24,4	0,2799642
1455898_x_at	Slc2a3	24,4	0,2799642
1442223_at	Enah	24,2	0,5161509
1417059_at	Krtcap2	24,2	0,2799642
1451988_s_at	Chmp4b /// LOC674706	24,2	0,2206964
1438644_x_at	Commd9	24,2	0
1436479_a_at	Dpp7	24,0	0,2206964
1440167_s_at	Lpp	24,0	0,1013274
